# Genome analysis and comparative genomics of a *Giardia intestinalis *assemblage E isolate

**DOI:** 10.1186/1471-2164-11-543

**Published:** 2010-10-07

**Authors:** Jon Jerlström-Hultqvist, Oscar Franzén, Johan Ankarklev, Feifei Xu, Eva Nohýnková, Jan O Andersson, Staffan G Svärd, Björn Andersson

**Affiliations:** 1 Department of Cell and Molecular Biology, BMC, Box 596, Uppsala University, SE-751 24 Uppsala, Sweden; 2 Department of Cell and Molecular Biology, Karolinska Institutet, Box 285, SE-171 77 Stockholm, Sweden; 3 Science for Life Laboratory, KISP, Tomtebodavägen 23A, 171 65 Solna, Sweden; 4 Department of Evolution, Genomics and Systematics, EBC, Uppsala University, SE-752 36 Uppsala, Sweden; 5 Department of Tropical Medicine, 1st Faculty of Medicine, Charles University, 128 00 Prague 2, Czech Republic

## Abstract

**Background:**

*Giardia intestinalis *is a protozoan parasite that causes diarrhea in a wide range of mammalian species. To further understand the genetic diversity between the *Giardia intestinalis *species, we have performed genome sequencing and analysis of a wild-type *Giardia intestinalis *sample from the assemblage E group, isolated from a pig.

**Results:**

We identified 5012 protein coding genes, the majority of which are conserved compared to the previously sequenced genomes of the WB and GS strains in terms of microsynteny and sequence identity. Despite this, there is an unexpectedly large number of chromosomal rearrangements and several smaller structural changes that are present in all chromosomes. Novel members of the VSP, NEK Kinase and HCMP gene families were identified, which may reveal possible mechanisms for host specificity and new avenues for antigenic variation. We used comparative genomics of the three diverse *Giardia intestinalis *isolates P15, GS and WB to define a core proteome for this species complex and to identify lineage-specific genes. Extensive analyses of polymorphisms in the core proteome of *Giardia *revealed differential rates of divergence among cellular processes.

**Conclusions:**

Our results indicate that despite a well conserved core of genes there is significant genome variation between *Giardia *isolates, both in terms of gene content, gene polymorphisms, structural chromosomal variations and surface molecule repertoires. This study improves the annotation of the *Giardia *genomes and enables the identification of functionally important variation.

## Background

*Giardia intestinalis *(syn *G. lamblia *and *G. duodenalis*) is a flagellated protozoan parasite that belongs to the diplomonad group, which includes both parasitic and free living species. During the course of evolution this organism has adapted to a parasitic life-style, which involves passing through an infectious cyst stage and subsequently into the vegetative trophozoite stage. *G. intestinalis *infects humans and a broad range of other mammals including wild and domestic animals, and causes diarrhea. The parasite spreads through the fecal-oral route and constitutes a serious health problem in developing countries, where it contributes to malnutrition and growth inhibition in children [1,2]. In industrialized countries, waterborne outbreaks of *G. intestinalis *continue to be a health concern in public water supplies and recreational waters. Highly resistant *G. intestinalis *cysts are able to survive from weeks to months in cold water, and the infectious dose can be as low as 10 cysts [3,4]. Many *G. intestinalis *infections do not give rise to symptoms and chronic carriers of *G. intestinalis *have been documented and these are sources of new infective cysts [5,6]. In addition to being an important mammalian pathogen *G. intestinalis *is an interesting model organism since it has been suggested to be a primitive, early-branching eukaryote. However, recently a number of eukaryotic features have been described in *G. intestinalis *[7]: a putative nucleolus has been identified [8]; a vestigial mitochondrial organelle called the mitosome has been confirmed [9]; and the prokaryote-like metabolic pathways that have been identified are most probably a result of horizontal gene transfer [10]. Even so, *G. intestinalis *is unusual by having two identical and transcriptionally active nuclei, no peroxisomes as well as a lack of a recognizable stacked Golgi-apparatus in vegetative cells [2]. The genome of *G. intestinalis *is small (the haploid size is ~11.7 Mb) compared to most sequenced eukaryotic genomes and was first described by Morrison et al [11]. Each nucleus of the best studied *G. intestinalis *isolate WB contains a diploid set of 5 major chromosomes that are inherited individually [12,13]. Substantial karyotype variability has been reported among *G. intestinalis *isolates, most likely caused by the hypervariable subtelomeric regions [14]. Compared to some other unicellular species, eg. *Trichomonas vaginalis*, *Trypanosoma cruzi*, and even the diplomonad *Spironucleus vortens*, the *G. intestinalis *genome has not undergone a massive repeat expansion, possibly due to evolutionary constraints that act upon the genome or because a large scale proliferation of mobile elements has not occurred. The genome of *G. intestinalis *is compact in terms of intergenic space and non-coding regions. Promoters and untranslated regions are minimal and only four introns have been identified [11], which is consistent with the features of a compact genome.

*G. intestinalis *has previously been considered to be a single species, mainly due to similarity in morphology between different isolates. However, based on the genetic variation between strains, *G. intestinalis *can be considered to be a species complex [15,16]. Seven assemblages (genotypes) have been confirmed as distinct evolutionary lineages, by enzyme electrophoretic pattern studies and phylogenetics [17,18]. Moreover, sequence analysis has been used to stratify isolates into these groups, mainly based on the genes *glutamate dehydrogenase*, *beta-giardin*, *elongation factor-1 alpha*, *triose phosphate isomerase *and *small subunit rRNA*. Furthermore, a growing number of studies have shown phenotypic differences regarding metabolism and biochemistry, growth rates (both *in vivo *and *in vitro*), drug sensitivity, pH preference, infectivity, susceptibility to the *G. intestinalis *dsRNA virus and clinical features [19,18]. However, no genetic factors guiding host specificity or adaptability have been identified so far, possibly due to the limited genetic tools available for *G. intestinalis*.

Assemblages A and B are the only assemblages known to infect humans, C and D infect dogs, group E infects hoofed animals, including cattle, sheep, goats, pigs, water buffaloes and mufflons, F infects cats and G is restricted to rodents. One recent study analyzed the genetic variation from a large number of assemblage A and B isolates [20]. It has been proposed that assemblages A and B are distinct species, with the species names *G. duodenalis *for assemblage A and *G. enterica *for assemblage B [16]. However, the degree of genetic variation between other assemblages than A and B is largely unknown.

Whole genome data is important to establish phylogenetic relationships, to identify genotype specific markers for epidemiological studies and offers a novel approach to understand the genetic diversity on the subspecies level. Furthermore, comparative genomics is a powerful tool to reveal insights into the evolutionary history of microbial pathogens and can provide information as to how host specificity and virulence is determined. The genome-wide genetic variation in the *G. intestinalis *species complex remains largely unexplored, so far only two isolates from two assemblages have been subject to whole genome sequencing. The first *G. intestinalis *genome to be sequenced was the WB isolate (assemblage A). The WB genome was found to be 11.7 Mb in size with 4889 annotated coding sequences, extremely low levels of allelic sequence heterozygosity (<0.01%) and no known identifiable virulence factors [11]. More than 50% of the annotated coding sequences showed no similarity to any protein sequence in public databases, but of the genes with a functional annotation many are likely to represent lateral transfers from bacteria or archaea [21]. We previously reported genome sequencing and analysis of the assemblage B isolate GS and comparative genomics to the WB genome revealed a well conserved core of genes, but also extensive genomic rearrangements [22]. The GS isolate was found to have an abundance of allelic sequence heterozygosity as detected by high throughput sequencing. Moreover, a comparison of three genetically divergent *G. intestinalis *isolates allows improved annotation of protein coding genes and non-coding RNAs.

To further understand the biological differences between human- and non-human infective isolates of *G. intestinalis *we here present a draft genome sequence and analysis of the assemblage E isolate P15, originally isolated from a piglet in the Czech Republic. This is the first whole genome analysis of a field isolate of *G. intestinalis *with minimal cultivation in the laboratory. We therefore expect it to closely reflect a wild-type *G. intestinalis *isolate.

## Results

### Genome assembly and allelic sequence heterozygosity

Clustering of the shotgun reads (Table 1, Additional file 1) and manual finishing of the assembly resulted in 820 contiguous sequences (contigs) with an N50 of 71,261 bp and an average genome coverage of 47×. The relatively large number of contigs is likely to be caused by the presence of genomic repeats rather than areas of insufficient sampling. The combined contig length was 11,522,052 bp. The haploid genome size is likely to be somewhat larger, due to the collapse of certain repeat regions.

**Table 1:** Sequence data summary

	FLX	Titanium
**Read count**	698 868	940 272

**Data amount (Mbp)**	143	474

**Average read length (bp)**	205	504

The G + C content of the overall assembly is 47%, which is identical to GS but differs slightly from WB (49%). The lower G + C content in P15 and GS is most likely explained by non-perfect assembly of G + C rich repeat regions, such as VSPs or High Cysteine Membrane Proteins in the whole genome shotgun data sets. To circumvent the problem of different degree of assembly completion, we examined the G + C content of conserved syntenic genes (1:1:1 orthologs) and found the G + C content to be 46% (P15), 47% (GS) and 49% (WB). Orthologs in P15 have the lowest average G + C content while WB have the highest.

Only 255 positions of 10,704,988 analyzed positions passed the criteria for allelic sequence heterozygosity. Most heterozygous positions were located in short contigs and may therefore represent artifacts, for example chimeric contigs, rather than a real heterozygosity. None of the heterozygous positions were located in genes that have previously been used for genotyping.

The assembly contains 61 frame shifted genes, 48 of which were found to be interrupted by single base insertion or deletion errors which is consistent with the homopolymer sequencing errors related to the 454 technology. Six genes were present in regions where the assembly is of low quality. Five frame shifted genes could not be attributed to homopolymer sequencing errors or low coverage in the assembly and were designated as putative pseudogenes, these are described further below (Additional file 2).

### Genome annotation and prediction

**Figure 1:**
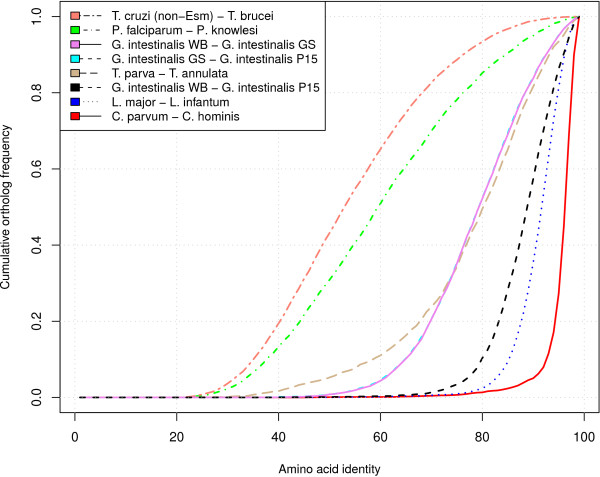
**A multi-species comparison of *Giardia *to other protozoan pathogens**. A comparison of the divergence between different species of protozoan pathogens. The following pairs of species were compared: *T. cruzi *(non-Esmeraldo) - *T. brucei*; *P. falciparum - P. knowlesi*; *G. intestinalis *WB - *G. intestinalis *GS; *G. intestinalis *GS - *G. intestinalis *P15; *T. parva *- *T. annulata*; *G. intestinalis *WB - *G. intestinalis *P15; *L. major *- *L. infantum; C. parvum - C. hominis*. The vertical axis shows the cumulative frequency of the ortholog count and the horizontal axis shows the corresponding amino acid identity. A steeper curve to the right indicate that the species pair is more closely related compared to a species pair to the left. In this comparison, *C. parvum *and *C. hominis *are the closest related species, whereas *T. cruzi *and *T. brucei *are the most distantly related based on protein similarity. *G. intestinalis *P15 and WB have a divergence close to what can be observed between *L. major *and *L. infantum*. *G. intestinalis *WB and GS have a divergence close to what is observed between *T. parva *and *T. annulata*. The ortholog relationships were established using reciprocal BLASTP and the protein identities were extracted from the BLAST outputs. Sequence data was downloaded from EuPathDB.

The vast majority of the P15 annotations were assigned using the information from the WB and GS genomes ([GenBank:AACB00000000] and [GenBank:ACGJ00000000]). The P15 draft genome sequence was found to contain 5012 annotated protein coding sequences, of which 2470 were conserved hypothetical genes. About 91% of the annotated genes had orthologs in WB. Genes without assigned orthologs were located in genomic regions without conserved synteny compared to the previously published *Giardia *genomes. The average protein identity between P15 and WB was 90% (sd. 7) and for the corresponding nucleotide sequences the identity was 87% (sd. 4). Only 15% of the ortholog pairs showed a nucleotide identity higher than 90%. The average protein identity between P15 and GS was 81% (sd. 10) and the nucleotide identity 77% (sd. 5). The higher average protein identity confirmed earlier phylogenetic studies [23] that showed assemblage E to be more closely related to assemblage A than assemblage B to A. Furthermore, 82% of the P15 genome assembly was identified as coding. The divergence between P15 and WB is close to what is observed between *Leishmania major *and *L. infantum *and the divergence between GS and WB is similar to that observed between *Theileria parva *and *T. annulata *(Figure 1).

### The *Giardia *core proteome and polymorphisms

The *Giardia *gene content can be subdivided into a core set of housekeeping genes and structural genes (approximately 91% of the *Giardia *genes), unique assemblage-specific genes and a more variable part of the genome that contains members of large gene families (approximately 9% of the *Giardia *genes; Additional file 3). The core genes contain conserved positional orthologs between P15, WB and GS. The ratio of non-synonymous (dN) and synonymous (dS) nucleotide changes was used to estimate the selective pressure on genes. In two comparisons (GS-WB and P15-GS) synonymous changes were found to be saturated and the ratio is not reliable. P15 and WB orthologs (see Methods) were used to calculate dN/dS in order to evaluate if certain genes have a differential evolutionary rate in *Giardia*.

**Figure 2:**
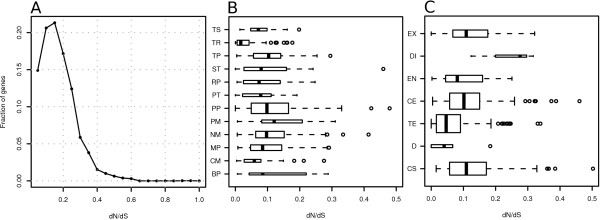
**Evolutionary analyses of *Giardia *genes compared between assemblage A and E**. The evolutionary rate of *Giardia *genes between P15 and WB was examined using estimates of dN/dS. Genes were grouped into gene ontology (GO) categories. The boundaries of each box plot represents the lower quartile (Q1 = 25%) and the upper quartile (Q3 = 75%) and the median value is displayed by the black line. The box width is proportional to the number of genes in the group. Statistical outliers are represented by circles. (A) The distribution of dN/dS estimates for genes with ortholog relationships between P15 and WB. The vertical axis shows fraction of the genes with a certain dN/dS ratio (horizontal axis). The curve is highly left skewed, indicating that most genes are under purifying selection. (B) dN/dS estimates for genes grouped after GO categories. The GO categories are: Transcription (TS); Translation (TR); Transport (TP); Signal transduction (ST); Regulation of biological process (RP); Protein Transport (PT); Protein metabolic process (PP); Primary metabolic process (PM); Nucleic acid metabolic process (NM); Metabolic process (MP); Carbohydrate metabolic process (CM); Biosynthetic process (BP). Five GO categories were significantly different using a Mann-Whitney test when compared to all other genes: translation; protein metabolic process; nucleic acid metabolic process; primary metabolic process; transport. (C) Distribution of dN/dS estimates in six *Giardia *developmental stages and one category for constitutively expressed genes. The category labels represent: EX = Excystation; DI = Differentiation; EN = Encystation; CE = Cyst and excystation; TE = Tropohozoite and encystation; D = Genes down-regulated during cyst and excystation; CS = Constitutively expressed genes. Four categories were found to be significantly different when compared to all other groups using a Mann-Whitney test: constitutively expressed genes; trophozoite/encystation; cyst/excystation; differentiation. The categorization is based upon publicly available SAGE data (GEO GSE8336).

The distribution of dN/dS values displayed a left skew with a peak around 0.15 (Table 2, Figure 2A), implying that most genes are under purifying selection. Some 104 genes were found to be perfectly conserved in pairwise comparisons between the three genomes when comparing on the amino acid level (Additional file 4). Among the genes with the highest dN/dS appeared uncharacterized genes, possibly due to a relaxation of the selective pressure or because of their importance in parasite adaptability. Using a slimmed gene ontology we mapped 981 *Giardia *genes to 12 broad categories (Figure 2B), which represent about 20% of the core gene content. The dN/dS distributions were grouped according to biological process GO category and the categories were found to be different from each other using a Kruskal-Wallis test. Five GO categories were significantly different using a Mann-Whitney test when compared to all other genes: protein metabolic process; nucleic acid metabolic process; primary metabolic process; translation; transport.

**Table 2:** Comparative genomics data

	P15-WB	P15-GS	WB-GS
**Orthologs**^a^	4564	4180	4301

**Protein identity**	90% (sd. 7)	81% (sd. 10)	78% (sd. 14)

**dN**^b^	0.06 (sd. 0.06)	0.13 (sd. 0.09)	0.13 (sd. 0.08)

**dS**^c^	0.44 (sd. 1.48)	1.33 (sd. 2.18)	1.30 (sd. 2.15)

**dN/dS**	0.15 (sd. 0.12)	0.11 (sd. 0.09)	0.11 (sd. 0.09)

^a ^Orthologs refer to positional orthologs. Genes truncated in contig ends are not included.

^b ^The rate of nonsynonymous and nucleotide variation. Calculated using the program yn00.

^c ^The rate of synonymous and nucleotide variation. Calculated using the program yn00.

Publicly available Serial Analysis of Gene Expression (SAGE) data was used to group *Giardia *genes into 6 developmental categories and one category for constitutively expressed genes (Figure 2C). Hierarchical clustering of data from 10 SAGE libraries have been used to construct the following developmental categories: excystation; differentiation; encystation; cyst/excystation; trophozoite/encystation; cyst/excystation genes that are downregulated; constitutively expressed genes [24]. The distribution of dN/dS ratios was examined and the groups were found to be different from each other using a Kruskal-Wallis test. Four categories were found to be significantly different when compared to all other groups using a Mann-Whitney test: constitutively expressed genes; trophozoite/encystation; cyst/excystation; differentiation. The differentiation group contain the smallest number of genes (n = 3).

Small insertion-deletion (indel) events were found to be abundant between orthologs. We produced three-way alignments of orthologous genes in GS, P15 and WB in order to identify lineage specific adaptions. Alignment gaps were interpreted as indel events along the branches of the phylogenetic tree ((P15, WB), GS). In order to find lineage specific indels, we defined an indel event as an alignment gap in one lineage that did not occur in the other two lineages, or correspondingly that one lineage lacked an alignment gap but a gap of the same length and start position was present in the two other lineages. The requirement of the same gap length and start position was implemented since gaps of different lengths would be caused by more than one indel event. Furthermore, a quality filter was implemented where 15 bp downstream and upstream of the genes was examined for mismatches and if more than 7 mismatches were identified the indel was rejected. Indels located in non-coding sequences were not considered because of the risk of inaccurate alignments. Some 515 indels were found to be specific for the P15 lineage (196 insertions and 319 deletions). The median deletion length was 3 bp and the median insertion length 6 bp. The most common length of an indel was 3 bp and the longest indel was 75 bp. Some 738 indels (303 insertions and 435 deletions) were found for the WB lineage.

### Differential distribution of genes

**Figure 3:**
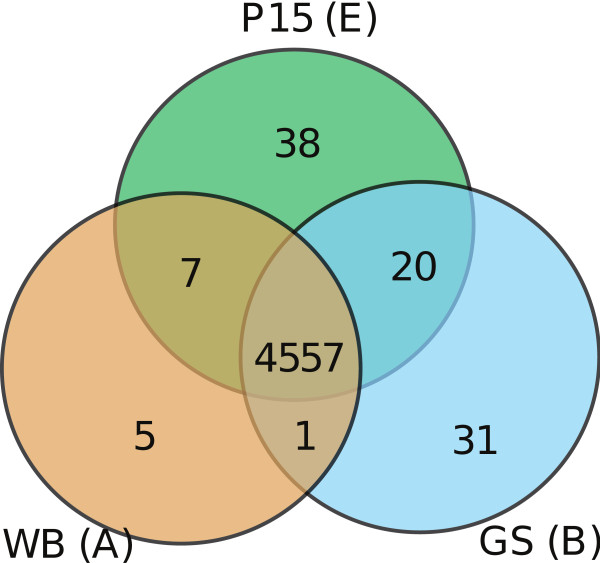
**A comparison of the shared and nonshared gene content**. The Venn diagram shows the shared and nonshared gene content of the isolates WB (assemblage A), GS (assemblage B) and P15 (assemblage E). The intersection displays the shared gene content between 2 or 3 genomes. Orthologs were defined using reciprocal BLAST and synteny information, as outlined in Methods. Specific genes were identified using BLASTP and TBLASTN searches with subsequent inspection of search results. Members of large gene families were not included in this comparison. The core gene content of *Giardia *consists of 4557 genes. Some 38 genes were found to be specific for P15, 5 genes for WB and 31 genes for GS. WB and P15 share 7 genes that are not present in GS, whereas GS and P15 share 20 genes that are not present in WB. WB and GS only share one gene which is not present in P15.

Comparisons between the three *Giardia *isolates revealed the presence of genes specific for each isolate (Figure 3, Additional file 2). In terms of specific genes, the P15 isolate has a few more (38) than GS, while the WB isolate has the smallest subset (5). The identified genes are not members of the large *Giardia *gene families and most of them have no sequence similarity to known sequences and their functions are therefore unknown. One P15 specific gene (GLP15_874) encodes an acetyl transferase. BLAST searches indicated high similarities to bacterial homologs of the gene. A phylogenetic analysis showed that this P15 gene clusters with bacterial sequences, mostly from a low G+C gram positive group (Firmicutes) suggestive of a recent gene acquisition (Additional file 5). The donor lineage cannot be determined due to poor bootstrap support values and likely frequent transfer of the gene among bacteria. Nevertheless, many of the putative donor lineages (i.e. *Lactobacillus*, *Cloststridium*, *Anerotruncus *and *Enterococcus*) are common inhabitants of the gastrointestinal tract of mammals (Additional file 5). This is consistent with a recent uptake of the gene from intestinal bacteria.

In this comparison, 31 GS specific genes were identified, and several conserved bacterial domains were identified in these genes as reported previously [22]. P15 and WB share only 7 genes that are not present in GS (Figure 3). In comparison, WB and GS share only one specific protein whereas P15 and GS share 20. Thirteen of these genes are located in a 20 kb contig in P15. Homologous genes were identified in GS on several small contigs. Synteny analysis of this chromosomal region in the WB genome indicates that a rearrangement has occurred when compared to P15. Preliminary data indicates that this region may also be absent from other *Giardia *assemblage A isolates. One of the genes only present in P15 and GS (GLP15_2168) contains domains similar to a prokaryotic DNA segregation protein and a second contains a possible nucleoside triphosphate hydrolase domain. However, the function of these proteins remains unknown. We detected the presence of 16 candidate pseudogenes in the *Giardia *genomes, 5 that were detected in WB and 6 in GS (Additional file 2). All of these are members of common gene families and all but one have positional orthologs in the GS-P15 and P15-WB genome comparisons, respectively. The remaining 5 pseudogenes were identified in P15. Candidate pseudogenes in WB and P15 were confirmed to have disrupted coding sequences by sequencing of PCR products (data not shown). SAGE-tags were not detected for WB pseudogenes, but could be detected for some WB orthologs to the confirmed pseudogenes in P15 and putative pseudogenes in GS (Additional file 2). One pseudogene in P15 involves a lateral transfer candidate (Additional file 5). Intact orthologs are present in the human associated *G. intestinalis *WB and GS stains, and only four bacterial strains from three different groups. Thus, the gene has a very patchy phylogenetic distribution in the tree of life. Some of the bacteria that do encode the gene are associated with mammals; *Collinsella aerofaciens *is part of the normal gut flora in humans, and *Ruminooccus *inhabit the rumen of cattle.

### Small RNAs and regulation of gene expression

The prediction of transfer RNAs (tRNA) was performed using tRNAscan-SE [25]. The WB genome contains 63 tRNA genes, two of which were predicted to be pseudogenes [26]. In P15, 61 tRNA genes were predicted and 5 of them contained an intron. In contrast, 58 tRNAs were identified in the GS genome and 7 of these contained an intron. The three genomes contain the same complement of tRNA genes but the copy number of each gene can be variable.

The same four introns were identified in the *Giardia *WB and GS genomes [22] and introns were found in the same positions in the P15 genome. Regular expressions constructed from consensus sequences of all known *Giardia *introns failed to locate additional candidates. The encystation inducible promoters from P15 resemble those of WB. Binding sites for the transcription factor Myb2 can be identified in the equivalent positions in the promoters of the cyst wall protein-1, cyst wall protein-2 and Myb2 itself. P15 harbors Myb2 binding sites in the sugar synthesis genes *glucosamine-6-phosphate isomerase *and *UDP-glucose 4-epimerase *as in WB, a distinguishing feature that is missing in the GS isolate.

Non-coding RNAs are generally well conserved, with P15-WB pairs showing the greatest similarity (Additional file 6).

### Variable *Giardia *gene families

Outside the core gene set, four highly variable gene families, the Variant-specific Surface Proteins (VSP), NEK Kinases, Protein 21.1 and High Cysteine Membrane Proteins (HCMP) predominate. The *Giardia *genomes analyzed so far contain approximately 200 each of the VSP and NEK Kinase genes. VSPs are the main parasite antigen in *Giardia *and these proteins have a conserved membrane spanning domain with an invariant cytoplasmic tail and a cysteine rich variable domain that is exposed to the extracellular environment. The NEK Kinases belong to the NIMA-related (Never In Mitosis Gene A) clade of serine/threonine kinases. Surprisingly, 76% of the NEK kinases in the *Giardia *WB genome are predicted to be catalytically inert.

We identified 112 complete VSP genes in the P15 genome assembly. However, there is also a significant number of VSP sequences that are present in small contigs in spite of the high coverage and relatively long read lengths. The average identity of VSPs between P15 and GS is 61% ± 10 which is slightly higher than for WB and GS. A search for the conserved terminal CRGKA motif in all the reads gave 4757 hits. With an average sequence coverage of 47× we estimated the number of VSP genes to be approximately 100, which is slightly lower than the number of annotated VSP genes. The reason could be incorrectly annotated VSP genes in shorter redundant contigs or possibly local variation in sequence depth. When we corrected for this, the VSP content is still lower in P15 compared to both WB and GS. VSP genes are rarely located in syntenic position in the genomes. This shows the plasticity of VSP genes both from the sequence perspective and from genomic location. The VSPs in P15 appear to be scattered throughout the genome as in WB and GS. Regions where VSPs and other dynamic protein families are found are characterized by significant differences in codon usage and higher G + C content. These regions are also frequently associated with breaks in synteny and rearrangements. The only regulatory motif that is uniquely associated with VSP genes in WB and P15 is the extended polyadenylation signal. This motif is remarkably well conserved over a stretch of 16 nucleotides (ACTTAGGTAGT[AG]AA[CT]GC) and is invariably located 0-15 bp downstream from the stop codon of VSP genes. Subtle differences in the consensus sequences can be distinguished between the isolates (Additional file 7). This sequence is also found in the small subset of VSP genes available from the GS isolate. Motif searches conducted on sequences upstream of the VSP genes revealed very few conserved sequences.

Phylogenetic analysis of VSP genes from P15 and WB was conducted using protein sequence alignment and maximum likelihood. The analysis indicated that VSP genes are distributed in two major subgroups in this tree which we designate as A and B, based on their distance in the phylogenetic tree (Additional file 7). The B subgroup is considerably smaller than the A subgroup but both subgroups contain both P15 and WB sequences. Certain branches of each subgroup show enrichment of either P15 or WB VSPs, which could reflect recent VSP duplication events. It is not clear whether the enrichment of WB genes is due a possible lack of assembled sequences for P15 or if this actually represents lineage specific diversification.

### Structural variation in *Giardia *genomes

Structural variation analyzed across the three *Giardia *genomes revealed frequent loss of genome synteny. In total, 141 cases of insertions, deletions and rearrangements could be detected. In most cases, the exact type of rearrangement could not be determined due to the termination of contigs in P15 and GS and occasionally in WB. Only 28 synteny breaks were recorded between WB and GS in contrast to 113 instances in the phylogenetically more related WB and P15. This discrepancy can be explained by the more fragmented GS draft assembly in which very few genes from repetitive gene families could be assembled. The deep coverage and overall quality of the P15 assembly increases the amount of structural variants that can be detected. We investigated the quality of our assembly by PCR verification of 10 structural variants (Additional file 8). This set included predicted intra- and inter-chromosomal rearrangements involving all chromosomes except chromosome 2. Seven out of 10 synteny breaks yielded the expected PCR product, including potential inter-chromosomal rearrangements between chromosomes 1 and 5 as well as 3 and 5. Intra-chromosomal events were verified on chromosomes 3, 4 and 5. The percentage of verified synteny breaks is higher in the P15 assembly (70%) than what was found experimentally in the GS draft genome (59%) [22].

**Figure 4:**
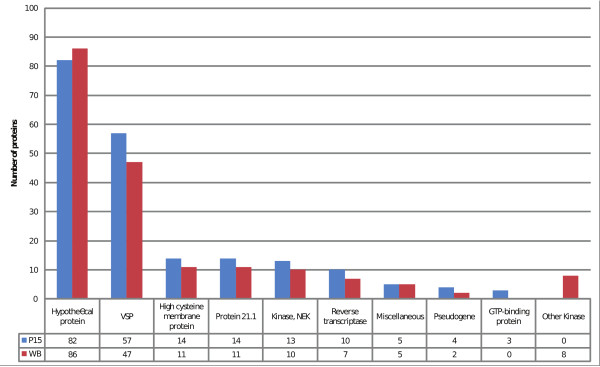
***Giardia *genes in non-syntenic regions**. Genes associated with non-syntenic genomic regions in P15 and WB were categorized according to their annotation. The number of genes in each category is displayed on the vertical axis. The profiles of genes are strikingly similar between isolates. Hypothetical genes and VSPs are the most common gene category found associated with non-syntenic genomic regions followed by the large *Giardia *gene families High cysteine membrane proteins, Protein 21.1 and NEK Kinases. The number of Kinases in unique regions is higher in WB whereas GTP-binding proteins are more frequent in P15.

Genes found in non-syntenic regions of the genomes were categorized according to annotation and the number of genes in each category were compared for WB and P15 (Figure 4). Hypothetical proteins was the most common category (WB: 86, P15: 82) followed by VSPs (WB: 47, P15: 57). Protein 21.1, HCMPs, NEK Kinases and putative transposable elements were also found to be associated with synteny breaks in these *Giardia *genomes. Large fractions of genes belonging to these gene families have diverged significantly, indicating that these gene families evolve more rapidly than other genes. Genes for GTP-binding protein and reverse transcriptase were more commonly associated with synteny breaks in P15 whereas Kinases were found more often in WB. Retro-transpososon associated sequences such as reverse transcriptases or endonucleases are found 17 times in 13 distinct non-syntenic positions in P15 (10 instances/8 distinct loci) and WB (7 instances/5 distinct loci).

Indications of the highly variable genome structure in *Giardia *have been noted previously in several reports [14,27-29]. Subtelomeric regions containing ribosomal DNA repeats are prone to rearrangements and copy number variation whereas the central region of chromosomes appear to be comparatively stable. The *Giardia *rearrangement rate of rDNA gene clusters has been estimated to be 1% per cell division *in vitro *[30]. Differences in chromosome number of individual *Giardia *nuclei have been reported for *in vitro *cultivated isolates. Five distinct chromosomal linkage groups have been demonstrated in spite of the karyotype diversity seen in *Giardia*. The karyotype heterogeneity could result from recombination associated with the presence of mobile elements. The WB genome contains three families of retrotransposons, two of which are located at the telomeres and could still be active [31].

**Figure 5:**
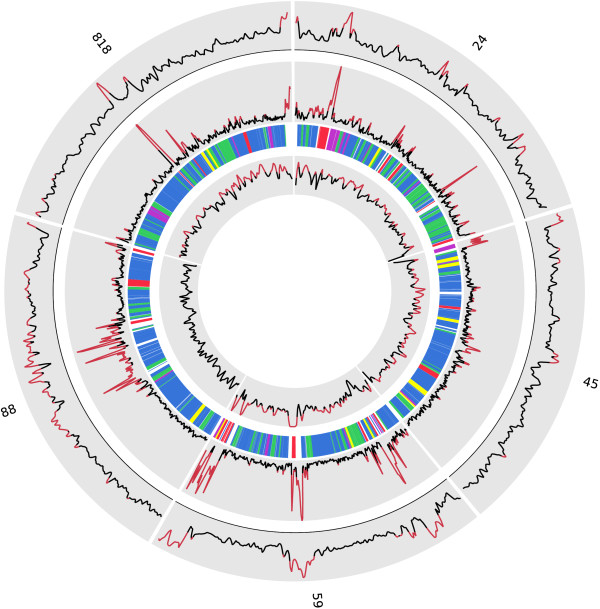
**A circular overview of five *Giardia *P15 contigs**. A circular overview of the five largest contigs in the P15 assembly which in total represent 909 kb of the genome (about 8%). The most outer graph displays G + C content plotted in overlapping 1.5 kb windows along the contigs. Areas where the G + C content is elevated (>50%) is displayed in red. The middle graph displays atypical nucleotide composition (as defined in Methods), plotted in overlapping 1.5 kb windows. Radial lines represent genes, with different colors for different annotations (blue = Hypothetical protein, red = Variant-specific surface protein (VSP), Reverse transcriptase (RT) or High cysteine membrane protein (HCMP), yellow = NEK Kinase, purple = Protein 21.1, green = other). The most inner graph displays the level of sequence coverage. VSP, RT and HCMP genes are characterized by a higher than average G+C content and atypical nucleotide signatures. These genes are dispersed and create chromosomal islands with higher than average G + C.

VSPs were frequently found in non-syntenic positions in WB and P15. Genome rearrangements have been identified as crucial components in the generation of antigenic variation in other parasites. The mobile nature of VSPs observed here is likely to be involved in generating VSP diversity through gene duplication and recombination. The large-scale architecture of *Giardia *chromosomes seem to consist of gene rich regions displaying stable codon usage and conserved gene order interspersed by chromosomal locations with atypical codon usage (Figure 5). VSPs and HCMP genes contribute to create such chromosomal islands with higher than average G + C content and atypical nucleotide signatures.

Several smaller rearrangements were found in areas with well-conserved genes. These include insertion or deletion of individual or multiple genes, inversions and translocations (Additional file 9).

## Discussion

We have previously reported a draft genome sequence and analysis of the *Giardia *assemblage B isolate GS [22]. We here describe the first genome analysis of a non-human *Giardia *isolate, that of assemblage E isolate P15. We have chosen to use a high throughput strategy to sequence the genome of P15, taking advantage of the low cost of 454 sequencing and the rapid data generation. We have examined the genetic diversity of a mostly uncharacterized assemblage of *Giardia*, and identified intraspecific variation that can be used for population studies or genotyping in a clinical setting. Comparative genome analysis of the assemblage A isolate WB and the B isolate GS previously revealed evidence of chromosomal shuffling, an abundance of allelic heterozygosity (ASH) and approximately 900 conserved open reading frames that were left unannotated in the first *Giardia *genome project.

We have estimated the core gene content of *Giardia *to be approximately 91% of the total gene content, with the remaining 9% involved in antigenic variation and functions related to host specificity. A few members of the VSP and HCMP families were found to be positionally conserved between all three genomes, but the function of these syntenic surface proteins is not known. The *Giardia *genomes are relatively similar in terms of core gene content, but tend to differ with respect to synteny and in genes responsible for antigenicity. We have identified several small scale genomic changes, including insertions and deletions of coding and noncoding sequences. A small number of isolate specific genes were identified and in most cases these are located in nonsyntenic regions of the genome. As expected, the *Giardia *isolates have diverged repertoires of surface proteins (VSPs and HCMPs) primarily coded in nonsyntenic regions of the genomes. A similar genomic arrangement is present in the trypanosomes where syntenic regions are interspersed with large regions of surface molecule genes, noncoding sequences and repeats. The roles of such regions are unknown but could constitute regions of increased genomic plasticity and they could play a role in the generation of antigenic variation through recombination events. A chromosome-wide analysis of gene order have been hindered in our assembly by the lack of long range continuity, a result of the sequencing read length. Future efforts to characterize the *Giardia *spp. genomes would benefit from using paired-end libraries or other strategies to join repeated regions and to avoid the typical homopolymer errors associated with the 454 technology.

A very low level of allelic sequence heterozygosity was detected in the P15 genome, which contradicts findings in the GS genome, but is consistent with observations from the WB genome. The striking differences in ASH levels could imply different biology regarding sexual reproduction, GS could be a recent mixture of two isolates, or the presence of active meiotic components in either isolate. It has been suggested that the assemblage A isolates may have a mechanism to maintain low levels of ASH, and that such a mechanism could also be operating in some assemblage E isolates. However, low ASH levels in P15 does conflict with genotyping data from assemblage E isolates where double peaked chromatograms have been observed [32]. Low ASH may be a feature of this particular assemblage E isolate but the high abundance of double peaks from other isolates could also reflect the fact that mixed infections with different assemblage E subgenotypes are more frequent in animals. Further studies of additional assemblage E isolates are needed to resolve this issue. Several genes identified as involved in DNA repair and meiosis in *Giardia*, were previously shown to display larger than average sequence divergence as well as insertions and deletions in the GS isolate, compared to WB [22]. An analogous comparison with the P15 isolate reveals substantial divergence in the genes *Rad50*, *Rad52 *and *Mre11*, and GS specific insertions in *Smc5*, *Mlh1*, *Rad50 *and deletions in *Rad50*, *Msh6*, *Dmc1a *and *Smc1 *that are specific for the ASH-rich GS lineage. Only one insertion in *Rad50 *was specific for the WB lineage whereas none were detected in P15. However, it should also be noted that genes displaying a high degree of divergence in the GS lineage are also the most diverged when comparing WB and P15. Future studies of these differences correlated to the level of ASH in disparate isolates could reveal a mechanism for dealing with ASH accumulation. These differences in gene synteny and ASH between these three *Giardia *isolates suggest that there is ubiquitous inter-genotype genetic diversity.

Indels have previously been noted as a prominent feature of many *Giardia *proteins when compared to eukaryotic homologs. Small indels are an abundant feature in pairwise comparisons between all three genomes and they are dispersed over a major fraction of the genes. In contrast to single nucleotide polymorphisms (SNPs), indels always alter the primary amino acid sequence, and are therefore more likely to impact protein function. We found that a number of genes involved in core processes of the cell have indels, which could possibly alter the functional specificity or efficiency of these proteins. Indels have been described in other species and have been attributed to DNA polymerase slippage [33].

Several groups encompassing metabolic genes were found to have elevated dN/dS ratios (Figure 2B). Such changes could cause substrate specificity to change in enzymes or alter metabolic pathways. It is possible that these enzymes allow variation in the amino acid sequence without affecting protein function. Another possibility is that these enzymes evolve more rapidly due to adaptations to the intestinal microenvironment in the host organism. A similar differential pattern of dN/dS rate was observed for genes grouped according to developmental stage. We here observed that genes expressed in certain developmental stages have slightly different distributions of dN/dS values (Figure 2C). The reason for this could be that certain stages need to be more tightly regulated and cannot easily accommodate variation. Another possibility would be that some stages expose proteins to the external environment, which could cause selective constraints by the host immune system.

We found evidence for a highly conserved set of core genes in *Giardia *which we propose is essentially common to all *Giardia intestinalis *isolates. This set of genes lies in genomic regions with mostly conserved synteny. Despite this, polymorphisms in terms of synonymous and nonsynonymous SNPs and indels are common in this set of genes, which could change the function or specificity of the expressed proteins. Also, cases of disrupted synteny do occur in these regions, which indicate that chromosomal recombination takes place in *Giardia *or in certain lineages of the species. Experimental evidence for recombination between assemblages is missing, but has been reported to occur between A2 isolates [34].

A large fraction of genes in the diplomonad genome have been proposed to have been acquired via horizontal gene transfer, due to their clustering with bacterial sequences in phylogenetic trees [10,35,26]. Here we found a number of genes only present in certain isolates (Figure 3). Some of these genes could be involved in shaping phenotypic differences, whereas others could be non-functional remnants from recent horizontal gene transfers, or represent recent losses in one or two of the lineages. An example of a recently acquired gene is the acetyl transferase gene with an apparent bacterial origin which was identified in P15, but which is absent in WB and GS (Additional file 5). However, it is presently unknown if the gene is expressed and what the precise function of its putative product might be. Our gene content comparison (Figure 3) showed that gene acquisition and loss is much less frequent than in intestinal bacteria, such as *Escherichia coli *[36]. Nevertheless, it indicates that gene acquisition is an extant evolutionary process also in a eukaryotic parasite, which likely contributes to diversification of *Giardia *isolates over evolutionary timescales.

The dynamics of the *G. intestinalis *genomes are further exemplified by a putative pseudogene found in a conserved core region of assemblage E. Evolutionary analyses revealed that the gene most likely was recently acquired from bacteria, probably of the mammalian gut flora (Additional file 5). The gene then turned into a pseudogene in P15, whereas it is under purifying selection in WB and GS. The presence of this gene exclusively in the human infecting isolates is intriguing; it is tempting to speculate that it might be related to host specificity.

We could not find any clear evidence for chromosomal duplications in our genome data, but we detected the presence of a genomic region in P15 that harbors 13 genes that are absent in WB while it appears that they are present in GS. A preliminary analysis suggests that this region may be absent from other assemblage A isolates as well, and the protein products from these genes could constitute good targets for the development of assemblage discriminating antibodies for serological purposes. Host specificity or phenotypic variation could also manifest at the level of expression. Differential expression of genes between isolates could provide means for creating host specificity and phenotypic differences. The regulation of gene expression is likely to be mostly post-transcriptional because of the short relaxed promoters in *Giardia*, and it is possible that short RNAs could be involved. Future efforts to characterize the *Giardia *transcriptome would benefit from using high throughput sequencing to efficiently enhance the resolution and confirm the expression of proposed gene models.

The repeat content of the *Giardia *genome is approximately 9% depending on which gene families are taken into consideration. This can be compared to the 50% repeat content of another protozoan parasite, *T. cruzi *[37]. The relatively low complexity of the *Giardia *genome makes it ideal for rapid comparative genomics using shotgun sequencing.

Repeated areas in the *Giardia *genome contain pseudogenes, retro-transposons and low complexity repeats and they could constitute areas of increased genomic plasticity or sites where recombination occurs, which is indicated by their associations with discontinued synteny. Proteins that are encoded in these regions have no positional orthologs in other genomes but tend to be classified into one of the larger gene families in *Giardia*. In other genomes with short intergenic distances, gene order has mostly been conserved. Also, the bidirectional promoters of *Giardia *could act to prevent reshuffling by inactivating genes upon rearrangement. Whether genomic rearrangements occur randomly, or in a controlled manner by a certain mechanism or genomic location, warrant further investigation. It is known that co-regulated genes act as a hindrance to genomic rearrangements. It could therefore be proposed that the lack of gene regulation at the transcriptional level allows for larger genomic rearrangements in *Giardia *without affecting gene function. The presence of retro-transposon derived sequences in non-syntenic genomic positions in WB and P15 could indicate retro-transposons have contributed to shaping the *Giardia *genomes by increasing the genomic plasticity.

The *Giardia *genomes contain a very large fraction of hypothetical genes that code for proteins of unknown function and with limited expression or proteomic data to support their expression or function. Most of these genes appear to be randomly distributed across the genome. The lack of significant hits to other protozoan genomes likely reflects the strong adaptation of the parasite to its host environment in combination with the evolutionary divergence of these organisms. As the number of sequences from diplomonads increases, due to a number of ongoing diplomonad sequencing projects, several *Giardia *proteins will likely be assigned orthologs, but experimental efforts will be required to elucidate the function of these proteins. The generation of the GS and P15 draft genomes have provided information about the strong conservation of these genes which indicates that they have a functional role in *Giardia *biology.

It is clear that the genomic data alone cannot resolve the question of host specificity and mechanisms of pathogenicity, but the genome sequence data provide an evolutionary insight into how parasite genomes have been shaped over the course of evolution. Moreover, the identification of lineage specific genes and gene variants will provide candidates for future functional studies. We hope that the draft genome sequence of this assemblage E isolate will provide useful information for future studies of the differences between *Giardia *strains and the diplomonads in general.

## Conclusions

Our results indicate that there is extensive genetic variation between *Giardia intestinalis *lineages. This includes chromosomal recombination, single nucleotide polymorphisms and insertion-deletion events. The assemblages contain diverged repertoires of *Giardia *surface proteins and other gene families, including VSPs, HCMPs, Protein 21.1 and NEK Kinases, including hundreds of distinct proteins for each of the three sequenced *Giardia *genomes. The analysis of orthologs indicate a stable set of core genes, present in all of the examined assemblages, and only a small number of isolate specific genes have been identified. Certain categories of genes were found to be under a differential evolutionary rate. However, for a complete understanding of the genomic landscape of *Giardia *it would be useful to combine data from more than one sequencing platform to understand the chromosome-wide differences.

## Methods

### Reagents and cell culture

The P15 strain was originally isolated in the Czech Republic from the upper jejunum of a necropsied piglet that was naturally infected with *Giardia *[38]. Unless otherwise indicated, the reagents used for cell culture were obtained from Sigma Chemical Co, USA. *Giardia intestinalis *strain P15 trophozoites were grown in standard conditions. Trophozoites from *in vitro *passage 10 were used for DNA extraction. Genomic DNA was extracted using the Easy-DNA kit (Invitrogen, Carlsbad, CA, US, Cat. no. K1800-01) followed by purification on a G100 Genomic tip (Qiagen).

### Sequencing, assembly and annotation

The genomic DNA was sequenced using a Genome Sequencer FLX/Titanium instrument (454 Life Sciences). Preparation and sequencing of the sample was performed according to the manufacturer's instructions. Base-calling was performed using the bundled 454 software. Three runs with the Genome Sequencer (454 Life Science) generated 1,639,140 reads (Table 1, Additional file 1). Genome assembly was performed *de novo *using the sequence assembly software MIRA V2.9.43 with the parameters *-job = denovo,genome,normal,454 -SK:mnr = yes *[39]. Manual inspection and editing of the assembly was carried out using consed and custom Perl scripts. The assembly process was complicated by the presence of repeats, which were too long to be spanned by the relatively short 454 reads.

A contamination screen was performed on the assembly using the megablast program [40] against GenBank human and fungi genomic databases and a few suspicious short, low coverage contigs were removed. Possible mis-assemblies were identified using coverage analysis and split at these points. Contigs less than 500 bp were removed from the assembly together with contigs with an average coverage less than 10.

The quality of our draft sequence is determined by several factors: the read length; the frequency of sequencing errors; the repeat content; the presence of allelic sequence heterozygosity. Given the high coverage (47×) of the genome sequence, we expect the quality of the sequence to be high and substitution errors extremely rare, however, a few (< 100) indel errors were identified due to their presence in open reading frames. The quality of the final assembly was assessed by evaluation of the consensus quality scores, only 1.69% of all positions in the assembly have quality scores less than 30. According to Phred quality scores this equals a base calling accuracy of 99.9% or 1 error in 1000 bases [41]. This would equal to 20,974 incorrectly called bases or 0.16% of the combined length.

	The annotation and subsequent analysis of the contigs have primarily been focused on the non-repetitive parts of the genome. Gene prediction was performed using GLIMMER 3 and CRITICA [42,43]. Open reading frames (ORFs) with invalid start codons were removed together with short, overlapping ORFs. An automatic annotation was performed by pooling the ORFs from WB and GS and using the best reciprocal BLAST hit as a preliminary annotation. All annotations were manually curated by aligning the contigs against the scaffolds of WB and GS using TBLASTX.

Alignments of putative frame shifted genes (n = 61) were manually inspected using the assembly viewer Tablet (version 1.10.02.08) [44]. The open reading frame of 48 genes could be restored by manual inspection of the assembly alignments. Genes not fulfilling these criteria were either classified as being present in a problematic region (n = 6) or as being putative pseudogenes (n = 5) (Additional file 2).

Orthologs were assigned to homologous genes in syntenic position by using reciprocal BLAST searches and manual curation of the results. Ortholog alignments were created using the transAlign program which increases the accuracy of a nucleotide alignment by using the corresponding amino acid alignment as a template [45]. Whole genome alignments along with genomic features were visualized using the Artemis Comparison Tool (ACT) [46]. ORFs located in contig breaks that span two or more contigs were annotated as partial and designated herein as truncated. In order to improve the *Giardia *genome annotation, all hypothetical proteins were queried against the GenBank non-redundant protein database and the GenBank Conserved Domain Database (curated protein families from Pfam and other sources). Statistics were done using the R software platform (version 2.9.2). A Kruskal-Wallis test and a Mann-Whitney test were conducted to test significance of Gene Ontology categories. 5% was used as a significance level. SAGE data was used as present in Gene Expression Omnibus and Stekel et al.'s *R*-statistic was used to select differentially expressed genes (R > 8) and clustered according to expressional profile [24].

This Whole Genome Shotgun project has been deposited at DDBJ/EMBL/GenBank under the accession [GenBank:ACVC00000000]. The version described herein is the first version, ACVC01000000. The data is also available in GiardiaDB [47].

### Isolate specific genes and synteny

Isolate specific genes were identified by reciprocal BLAST searches, using BLASTP and TBLASTN (cutoff E = 1e-20). The final gene lists were compiled by manual curation of BLAST search results and investigation of the associated genomic regions. While the current assembly limited us from studying chromosome wide synteny, we analyzed synteny blocks and microsynteny. Genes associated with synteny breaks were recorded and categorized according to available annotation.

### Evolutionary analysis and allelic sequence heterozygosity

Calculation of the dN/dS ratio was done using the yn00 program of the PAML package [48] (version 4.2b). The trinucleotide composition was examined using χ^2 ^analysis of codon frequencies. This was performed by counting the frequencies of 3-mers in all 6 reading frames in overlapping windows and using the genome wide 3-mere frequencies as the expected value when calculating the χ^2 ^statistics. By plotting the data using Circos [49], distinct peaks appear in areas where the trinucleotide (atypical) composition deviate from the average.

Phylogenetic trees were constructed from clustalw2 protein alignments and clustered using maximum likelihood, as implemented in the software RAxML (version 7.0.4) [50]. The gamma model of rate heterogeneity was used together with the WAG substitution model for the optimal tree. Bootstrap analyses were performed on 100 replicates using the same settings. The VSP tree was rendered using the ape package for the R statistical platform [51].

We used strict criteria to define a single position as a possible allelic sequence heterozygosity (ASH). Only positions with higher coverage than 20× were analyzed without gaps or ambiguous nucleotides ('n'). Alternative nucleotides located in reads with identical start positions were ignored because of the likelihood of sequencing errors. Further, alternative nucleotides would be required to be present in at least 15% of the counted positions.

### Introns, non coding sequences and motif discovery

Introns in the P15 genome were identified by alignment of syntenic regions in the three *Giardia *genomes. Introns and surrounding sequences from the three *Giardia *genomes were extracted and aligned using ClustalW. The aligned sequences were manually edited and used to build a regular expression capable of extracting all known *Giardia *introns from the WB, GS and P15 *Giardia *genomes. We analyzed the upstream and downstream regions of VSP genes in P15 and WB for common motifs using the MEME program [52]. Non-coding RNAs in P15 were identified by BLASTN searches using gene models from WB.

### Confirmation of pseudogenes and synteny breaks

Primers were designed manually according to recommendations in the Phusion HotStart II polymerase instruction manual (*Tm *60°C and 22-25 bp in length) and synthesized by Sigma-Genosys. The targets were amplified in a mixture containing 1 × Phusion HF buffer with 1.5 mM MgCl2, 200 μM dNTPs, 0.5 μM of the forward and reverse primers, 40 ng template genomic DNA (either P15 or WB) and 0.8 U Phusion HS II DNA polymerase (Finnzymes) in a total volume of 40 μl. The reactions were incubated for 3 min at 98°C followed by (98°C for 15 sec, 60°C for 15 sec, 72°C for 30 sec/1 kb of expected amplicon) × 35 cycles and were subsequently held at 4°C. Amplicons were separated by 0.7% 1 × TAE agarose gel electrophoresis and visualized by ethidium bromide staining. The PCR products were purified using the QIAquick PCR purification kit according to the manufacturer's recommendations and eluted in 30 μl Buffer AE. The purified PCR products were sequenced with their respective forward and/or reverse primers at the Uppsala Genome Center using the BigDye^® ^Terminator v3.1 (Applied Biosystems) chemistry followed by capillary electrophoresis on an ABI3730XL sequencer (Applied Biosystems).

## Abbreviations

(CDS): Coding Sequence; (ORF): Open Reading Frame; (ASH): Allelic Sequence Heterozygosity; (SNP): Single Nucleotide Polymorphism; (INDEL): Insertion or Deletion;

## Authors' contributions

JJH and OF carried out the bioinformatic analyses and wrote the manuscript. JA participated in the genome annotation. JOA and FX participated in the evolutionary analyses. BA, SGS, EN and JOA conceived the study, and participated in its design and coordination. All authors read and approved the final manuscript.

## Supplementary Material

Additional file 1 **Read length distribution and assembly coverage**. (A) Distribution of read lengths as generated by the 454 Genome Sequencer FLX/Titanium. The read length in base pairs (bp) is on the horizontal axis and the read count is on the vertical axis. The plot displays a bimodal curve with one peak at 250 bp and another one at 500 bp, typical for each one of the two platform types used for sequencing. (B) Shows the number of assembly positions on the vertical axis with a certain level of coverage on the horizontal axis. The plot shows that the average coverage is around 47 times. Click here for file

Additional file 2 **Specific genes, shared genes and disrupted open reading frames**. Contains lists of isolate specific genes, genes shared between two isolates and putative pseudogenes. A list of artificially disrupted open reading frames in the P15 data is also included. Click here for file

Additional file 3 **List of *Giardia *non-core and core genes**. A list of non-core genes in the P15 genome and *Giardia *core genes. A few genes lack annotated orthologs due to an incomplete genome assembly. Click here for file

Additional file 4 **Conservation of meiosis, DNA repair and ultraconserved proteins**. Conservation of putative Meiosis and DNA repair proteins in WB, GS and P15. Protein identities are recorded as percent extracted from ClustalW alignments of orthologs in WB, GS and P15. Several major deletions or insertions that are lineage specific were recorded. Most of these were specific for the GS lineage. A list of ultraconserved proteins in the genomes is included. Click here for file

Additional file 5 **Evolutionary analyses of a recently acquired Giardia gene**. Evolutionary analyses of a recently acquired *Giardia *gene. (A) A maximum likelihood tree of an acetyl transferase gene (GLP15_874). Only representatives of the homologs showing the highest similarity to the *Giardia *sequences are included. The tree is based on 139 unambiguously aligned amino acid positions. (B) Alignment of conserved hypothetical proteins from *G. intestinalis *GS (GL50581_4454) and WB (GL50803_10192) to all detected bacterial homologs. A putative amino acid sequence from a *G. intestinalis *P15 pseudogene is included and the positions of frameshift and in-frame termination codons are indicated. (C) A maximum likelihood tree based on 137 unambiguously aligned amino acid positions in the alignment shown in (B). Accession numbers are shown in parentheses. Only bootstrap values >50% are shown in the trees. Unambiguously aligned regions were identified manually and removed. Representative homologs with high sequence similarity to these genes were selected using the BLAST-EXPLORER software. Click here for file

Additional file 6 **Non-coding RNA conservation**. Conservation of non-coding RNAs across the three *Giardia *genomes. Non-coding RNAs are generally well conserved, with P15-WB pairs showing the greatest similarity. Examples of ultraconserved non-coding RNAs across all genomes include GLsR2 and GLsR8. Click here for file

Additional file 7 **VSP extended polyadenylation signal and VSP phylogenetic tree**. (A) Comparative analysis of the extended polyadenylation sequence of VSP genes. The conservation of the VSP polyadenylation motif was investigated using the MEME software. Downstream regions (200 bp) of VSP genes were extracted and queried for the presence of conserved motifs. The VSP genes of all three *Giardia *isolates carries a very similar conserved motif that extends over at least 16 bp and is strictly positioned 0-15 bp from the translational stop codon. The P15 motif is more strictly conserved and carries a few more semi-conserved positions for a longer motif than the corresponding motifs in WB and GS. (B) A phylogenetic tree of VSP genes from P15 and WB. The VSP phylogenetic tree is based on a clustalw2 protein alignment of 156 VSP genes from WB and P15. VSP genes were selected for alignment by all-against-all BLASTP, and only VSP genes that showed at least 65% protein identity to the best-matching non-identical hit were used for the alignment (n = 156). In order to improve the alignment, sequences were trimmed (190 aa were removed from the 5' part of all sequences). Subsequent clustering was performed using maximum likelihood as implemented in the software RAxML version 7.0.4 using with the rapid bootstrap setting (-f a). The tree was rendered using the ape package for R. Black dots represent nodes with > = 50% bootstrap support. The gene id (genbank locus tag) is indicated at the tip of the branch. Blue labels represent genes from WB while black labels represent genes from P15. The tree revealed two primary groups (A and B), which are distinguished based on their distance in the tree. Click here for file

Additional file 8 **Synteny breaks and PCR primers**. Experimental verification of 10 examples of structural variation discovered in the P15 genome. Click here for file

Additional file 9 **Structural variation in the *Giardia *genomes**. Genome structural variation detected during synteny analysis of the P15, WB and GS genomes. Click here for file

## References

[B1] Goto R Panter-Brick C Northrop-Clewes CA Manahdhar R Tuladhar NR Poor Intestinal Permeability in Mildly Stunted Nepali Children: Associations with Weaning Practices and Giardia Lamblia Infection British Journal of Nutrition 2002 88 141 14910 1079/BJN2002599 10.1079/BJN2002599 12171055

[B2] Adam RD Biology of Giardia lamblia Clin Microbiol Rev 2001 14 447 7510 1128/CMR.14.3.447-475.2001 10.1128/CMR.14.3.447-475.2001 11432808 PMC88984

[B3] LeChevallier MW Norton WD Lee RG Occurrence of Giardia and Cryptosporidium spp. in surface water supplies Appl Environ Microbiol 1991 57 2610 2616 1822675 10.1128/aem.57.9.2610-2616.1991 PMC183628

[B4] Craun GF Calderon RL Craun MF Outbreaks associated with recreational water in the United States International Journal of Environmental Health Research 2005 15 24310 1080/09603120500155716 10.1080/09603120500155716 16175741

[B5] Wolfe MS Giardiasis Clin Microbiol Rev 1992 5 93 100 1735095 10.1128/cmr.5.1.93 PMC358225

[B6] Hellard ME Sinclair MI Hogg GG Fairley CK Prevalence of enteric pathogens among community based asymptomatic individuals Journal of Gastroenterology and Hepatology 2000 15 290 29310 1046/j.1440-1746.2000.02089.x 10.1046/j.1440-1746.2000.02089.x 10764030

[B7] Lloyd D Harris JC Giardia: highly evolved parasite or early branching eukaryote? Trends in Microbiology 2002 10 122 12710 1016/S0966-842X(02)02306-5 10.1016/S0966-842X(02)02306-5 11864821

[B8] Jiménez-García LF Zavala G Chávez-Munguía B Ramos-Godínez MDP López-Velázquez G Segura-Valdez MDL Montañez C Hehl AB Argüello-García R Ortega-Pierres G Identification of nucleoli in the early branching protist Giardia duodenalis Int J Parasitol 2008 38 1297 130410 1016/j.ijpara.2008.04.012 10.1016/j.ijpara.2008.04.012 18625508

[B9] Tovar J Leon-Avila G Sanchez LB Sutak R Tachezy J van der Giezen M Hernandez M Muller M Lucocq JM Mitochondrial remnant organelles of Giardia function in iron-sulphur protein maturation Nature 2003 426 172 17610 1038/nature01945 10.1038/nature01945 14614504

[B10] Andersson JO Gene transfer and diversification of microbial eukaryotes Annu Rev Microbiol 2009 63 177 19310 1146/annurev.micro.091208.073203 10.1146/annurev.micro.091208.073203 19575565

[B11] Morrison HG McArthur AG Gillin FD Aley SB Adam RD Olsen GJ Best AA Cande WZ Chen F Cipriano MJ Davids BJ Dawson SC Elmendorf HG Hehl AB Holder ME Huse SM Kim UU Lasek-Nesselquist E Manning G Nigam A Nixon JEJ Palm D Passamaneck NE Prabhu A Reich CI Reiner DS Samuelson J Svard SG Sogin ML Genomic minimalism in the early diverging intestinal parasite Giardia lamblia Science 2007 317 1921 192610 1126/science.1143837 10.1126/science.1143837 17901334

[B12] Bernander R Palm JE Svärd SG Genome ploidy in different stages of the Giardia lamblia life cycle Cell Microbiol 2001 3 55 62 10.1046/j.1462-5822.2001.00094.x 11207620

[B13] Adam RD Biology of Giardia lamblia Clin Microbiol Rev 2001 14 447 47510 1128/CMR.14.3.447-475.2001 10.1128/CMR.14.3.447-475.2001 11432808 PMC88984

[B14] Le Blancq SM Adam RD Structural basis of karyotype heterogeneity in Giardia lamblia Mol Biochem Parasitol 1998 97 199 208 10.1016/S0166-6851(98)00150-9 9879898

[B15] Andrews RH Adams M Boreham PF Mayrhofer G Meloni BP Giardia intestinalis: electrophoretic evidence for a species complex Int J Parasitol 1989 19 183 190 10.1016/0020-7519(89)90006-4 2722391

[B16] Monis PT Caccio SM Thompson RCA Variation in Giardia: towards a taxonomic revision of the genus Trends Parasitol 2009 25 93 10010 1016/j.pt.2008.11.006 10.1016/j.pt.2008.11.006 19135417

[B17] Lalle M Pozio E Capelli G Bruschi F Crotti D Cacciò SM Genetic heterogeneity at the beta-giardin locus among human and animal isolates of Giardiaduodenalis and identification of potentially zoonotic subgenotypes Int J Parasitol 2005 35 207 21310 1016/j.ijpara.2004.10.022 10.1016/j.ijpara.2004.10.022 15710441

[B18] Thompson RCA Monis PT Variation in Giardia: implications for taxonomy and epidemiology Adv Parasitol 2004 58 69 13710 1016/S0065-308X(04)58002-8 10.1016/S0065-308X(04)58002-8 15603762

[B19] Cacciò SM Thompson RCA McLauchlin J Smith HV Unravelling Cryptosporidium and Giardia epidemiology Trends Parasitol 2005 21 430 43710 1016/j.pt.2005.06.013 10.1016/j.pt.2005.06.013 16046184

[B20] Sprong H Cacciò SM van der Giessen JWB on behalf of the ZOOPNET network and partners Identification of Zoonotic Genotypes of Giardia duodenalis PLoS Negl Trop Dis 2009 3 e55810 1371/journal.pntd.0000558 10.1371/journal.pntd.0000558 PMC2777335 19956662

[B21] Andersson JO Sjögren AM Davis LAM Embley TM Roger AJ Phylogenetic analyses of diplomonad genes reveal frequent lateral gene transfers affecting eukaryotes Curr Biol 2003 13 94 104 10.1016/S0960-9822(03)00003-4 12546782

[B22] Franzén O Jerlström-Hultqvist J Castro E Sherwood E Ankarklev J Reiner DS Palm D Andersson JO Andersson B Svärd SG Draft genome sequencing of giardia intestinalis assemblage B isolate GS: is human giardiasis caused by two different species? PLoS Pathog 2009 5 e100056010 1371/journal.ppat.1000560 10.1371/journal.ppat.1000560 PMC2723961 19696920

[B23] Ey PL Mansouri M Kulda J Nohýnková E Monis PT Andrews RH Mayrhofer G Genetic analysis of Giardia from hoofed farm animals reveals artiodactyl-specific and potentially zoonotic genotypes J Eukaryot Microbiol 1997 44 626 635 10.1111/j.1550-7408.1997.tb05970.x 9435134

[B24] Birkeland SR Preheim SP Davids BJ Cipriano MJ Palm D Reiner DS Svärd SG Gillin FD McArthur AG Transcriptome analyses of the Giardia lamblia life cycle Mol Biochem Parasitol 2010 10 http://www.ncbi.nlm.nih.gov/pubmed/20570699 1016/j.molbiopara.2010.05.010Available: Accessed 29 June 2010 20570699 10.1016/j.molbiopara.2010.05.010 PMC2972195

[B25] Lowe TM Eddy SR tRNAscan-SE: a program for improved detection of transfer RNA genes in genomic sequence Nucleic Acids Res 1997 25 955 964 10.1093/nar/25.5.955 9023104 PMC146525

[B26] Morrison HG McArthur AG Gillin FD Aley SB Adam RD Olsen GJ Best AA Cande WZ Chen F Cipriano MJ Davids BJ Dawson SC Elmendorf HG Hehl AB Holder ME Huse SM Kim UU Lasek-Nesselquist E Manning G Nigam A Nixon JEJ Palm D Passamaneck NE Prabhu A Reich CI Reiner DS Samuelson J Svard SG Sogin ML Genomic Minimalism in the Early Diverging Intestinal Parasite Giardia lamblia Science 2007 317 1921 192610 1126/science.1143837 10.1126/science.1143837 17901334

[B27] Le Blancq SM Chromosome rearrangements in Giardia lamblia Parasitol Today (Regul Ed) 1994 10 177 179 10.1016/0169-4758(94)90021-3 15275464

[B28] Upcroft JA Healey A Upcroft P Chromosomal duplication in Giardia duodenalis Int J Parasitol 1993 23 609 616 10.1016/0020-7519(93)90167-W 8225763

[B29] Tůmová P Hofstetrová K Nohýnková E Hovorka O Král J Cytogenetic evidence for diversity of two nuclei within a single diplomonad cell of Giardia Chromosoma 2007 116 65 7810 1007/s00412-006-0082-4 10.1007/s00412-006-0082-4 17086421

[B30] Le Blancq SM Korman SH Van der Ploeg LH Spontaneous chromosome rearrangements in the protozoan Giardia lamblia: estimation of mutation rates Nucleic Acids Res 1992 20 4539 4545 10.1093/nar/20.17.4539 1408754 PMC334182

[B31] Arkhipova IR Morrison HG Three retrotransposon families in the genome of Giardia lamblia: two telomeric, one dead Proc Natl Acad Sci USA 2001 98 14497 1450210 1073/pnas.231494798 10.1073/pnas.231494798 11734649 PMC64710

[B32] Lebbad M Mattsson JG Christensson B Ljungström B Backhans A Andersson JO Svärd SG From mouse to moose: Multilocus genotyping of Giardia isolates from various animal species Vet Parasitol 2010 168 231 23910 1016/j.vetpar.2009.11.003 10.1016/j.vetpar.2009.11.003 19969422

[B33] Viguera E Canceill D Ehrlich SD Replication slippage involves DNA polymerase pausing and dissociation EMBO J 2001 20 2587 259510 1093/emboj/20.10.2587 10.1093/emboj/20.10.2587 11350948 PMC125466

[B34] Cooper MA Adam RD Worobey M Sterling CR Population genetics provides evidence for recombination in Giardia Curr Biol 2007 17 1984 198810 1016/j.cub.2007.10.020 10.1016/j.cub.2007.10.020 17980591

[B35] Andersson JO Sjögren AM Davis LAM Embley TM Roger AJ Phylogenetic analyses of diplomonad genes reveal frequent lateral gene transfers affecting eukaryotes Curr Biol 2003 13 94 104 10.1016/S0960-9822(03)00003-4 12546782

[B36] Welch RA Burland V Plunkett G Redford P Roesch P Rasko D Buckles EL Liou S Boutin A Hackett J Stroud D Mayhew GF Rose DJ Zhou S Schwartz DC Perna NT Mobley HLT Donnenberg MS Blattner FR Extensive mosaic structure revealed by the complete genome sequence of uropathogenic Escherichia coli Proc Natl Acad Sci USA 2002 99 17020 1702410 1702410.1073/pnas.252529799 10.1073/pnas.252529799 12471157 PMC139262

[B37] El-Sayed NM Myler PJ Bartholomeu DC Nilsson D Aggarwal G Tran A Ghedin E Worthey EA Delcher AL Blandin G Westenberger SJ Caler E Cerqueira GC Branche C Haas B Anupama A Arner E Aslund L Attipoe P Bontempi E Bringaud F Burton P Cadag E Campbell DA Carrington M Crabtree J Darban H da Silveira JF de Jong P Edwards K The genome sequence of Trypanosoma cruzi, etiologic agent of Chagas disease Science 2005 309 409 41510 1126/science.1112631 10.1126/science.1112631 16020725

[B38] Koudela B Nohýnková E Vítovec J Pakandl M Kulda J Giardia infection in pigs: detection and in vitro isolation of trophozoites of the Giardia intestinalis group Parasitology 1991 102 Pt 2 163 166 10.1017/S0031182000062442 1852483

[B39] Chevreux B MIRA sequence assembler http://sourceforge.net/apps/mediawiki/mira-assembler/

[B40] Zhang Z Schwartz S Wagner L Miller W A greedy algorithm for aligning DNA sequences J Comput Biol 2000 7 203 21410 1089/10665270050081478 10.1089/10665270050081478 10890397

[B41] Ewing B Green P Base-calling of automated sequencer traces using phred. II. Error probabilities Genome Res 1998 8 186 194 9521922

[B42] Delcher AL Harmon D Kasif S White O Salzberg SL Improved microbial gene identification with GLIMMER Nucleic Acids Res 1999 27 4636 4641 10.1093/nar/27.23.4636 10556321 PMC148753

[B43] Badger JH Olsen GJ CRITICA: coding region identification tool invoking comparative analysis Mol Biol Evol 1999 16 512 524 10331277 10.1093/oxfordjournals.molbev.a026133

[B44] Milne I Bayer M Cardle L Shaw P Stephen G Wright F Marshall D Tablet--next generation sequence assembly visualization Bioinformatics 2010 26 401 40210 1093/bioinformatics/btp666 10.1093/bioinformatics/btp666 19965881 PMC2815658

[B45] Bininda-Emonds ORP transAlign: using amino acids to facilitate the multiple alignment of protein-coding DNA sequences BMC Bioinformatics 2005 6 15610 1186/1471-2105-6-156 10.1186/1471-2105-6-156 PMC1175081 15969769

[B46] Carver TJ Rutherford KM Berriman M Rajandream M Barrell BG Parkhill J ACT: the Artemis Comparison Tool Bioinformatics 2005 21 3422 342310 1093/bioinformatics/bti553 10.1093/bioinformatics/bti553 15976072

[B47] Aurrecoechea C Brestelli J Brunk BP Carlton JM Dommer J Fischer S Gajria B Gao X Gingle A Grant G Harb OS Heiges M Innamorato F Iodice J Kissinger JC Kraemer E Li W Miller JA Morrison HG Nayak V Pennington C Pinney DF Roos DS Ross C Stoeckert CJ Sullivan S Treatman C Wang H GiardiaDB and TrichDB: integrated genomic resources for the eukaryotic protist pathogens Giardia lamblia and Trichomonas vaginalis Nucleic Acids Res 2009 37 D526 53010 1093/nar/gkn631 10.1093/nar/gkn631 18824479 PMC2686445

[B48] Yang Z PAML: a program package for phylogenetic analysis by maximum likelihood Comput Appl Biosci 1997 13 555 556 9367129 10.1093/bioinformatics/13.5.555

[B49] Krzywinski M Schein J Birol I Connors J Gascoyne R Horsman D Jones SJ Marra MA Circos: an information aesthetic for comparative genomics Genome Res 2009 19 1639 164510 1101/gr.092759.109 10.1101/gr.092759.109 19541911 PMC2752132

[B50] Stamatakis A Ludwig T Meier H RAxML-III: a fast program for maximum likelihood-based inference of large phylogenetic trees Bioinformatics 2005 21 456 46310 1093/bioinformatics/bti191 10.1093/bioinformatics/bti191 15608047

[B51] Paradis E Claude J Strimmer K APE: Analyses of Phylogenetics and Evolution in R language Bioinformatics 2004 20 289 290 10.1093/bioinformatics/btg412 14734327

[B52] Bailey TL Williams N Misleh C Li WW MEME: discovering and analyzing DNA and protein sequence motifs Nucleic Acids Res 2006 34 W369 37310 1093/nar/gkl198 10.1093/nar/gkl198 16845028 PMC1538909

