# Supplementary figures and images for: Genome analysis and comparative genomics of a Giardia intestinalis assemblage E isolate
Source: BMC Genomics. 2010 Oct 7;11:543. doi: 10.1186/1471-2164-11-543 (PMC3091692; doi:10.1186/1471-2164-11-543)

**A**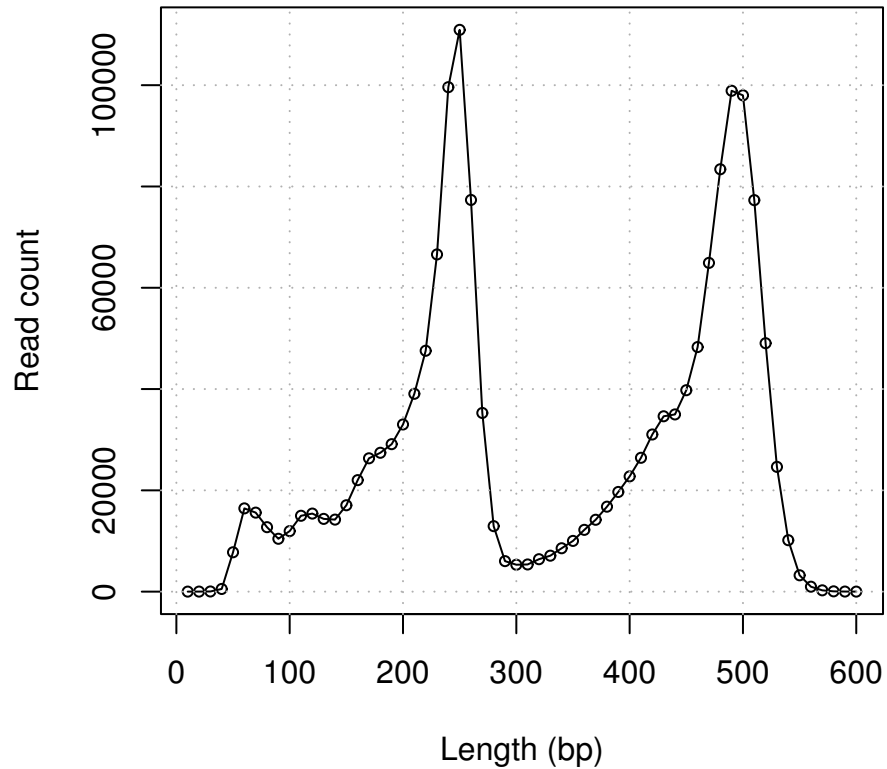**B**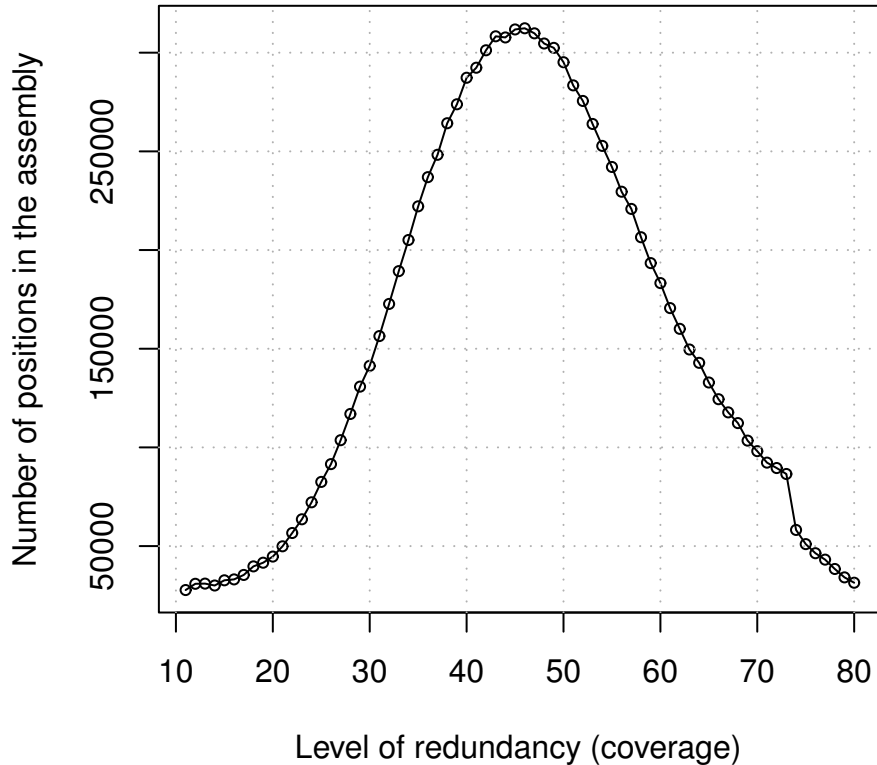

Supplement: Additional file 1 — Read length distribution and assembly coverage. (A) Distribution of read lengths as generated by the 454 Genome Sequencer FLX/Titanium. The read length in base pairs (bp) is on the horizontal axis and the read count is on the vertical axis. The plot displays a bimodal curve with one peak at 250 bp and another one at 500 bp, typical for each one of the two platform types used for sequencing. (B) Shows the number of assembly positions on the vertical axis with a certain level of coverage on the horizontal axis. The plot shows that the average coverage is around 47 times. [file 1471-2164-11-543-S1.PDF]

A

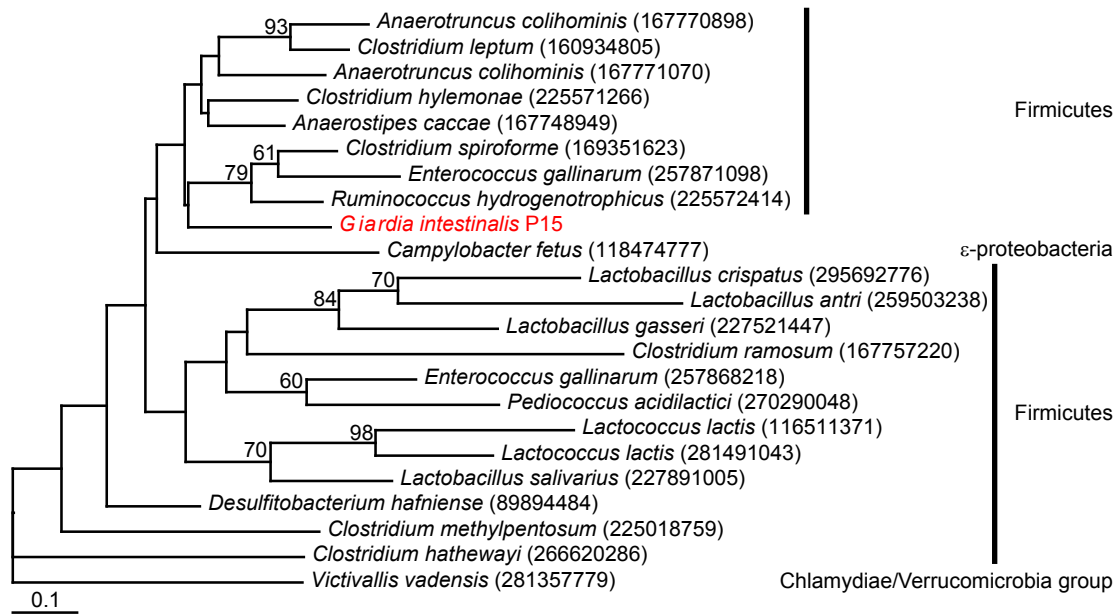

B

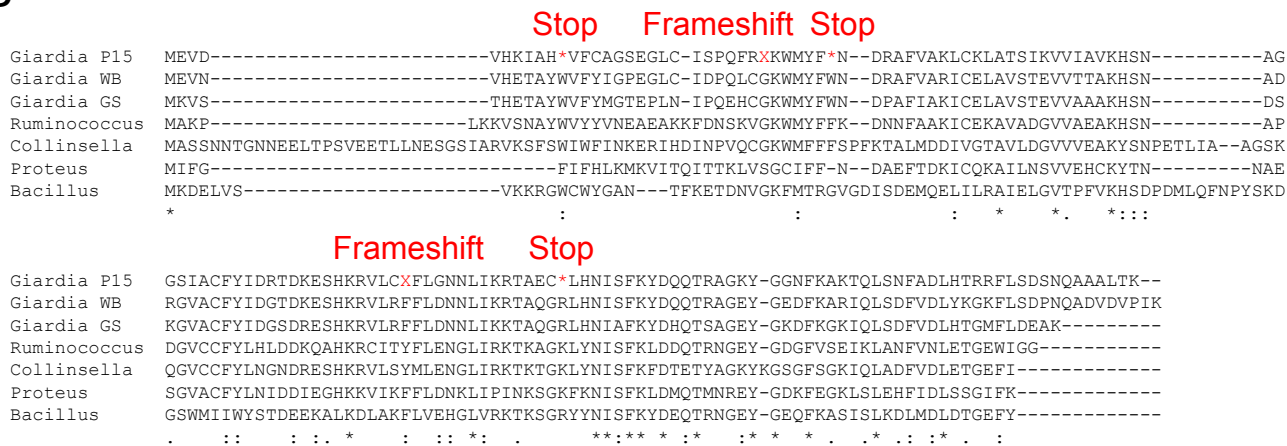

C

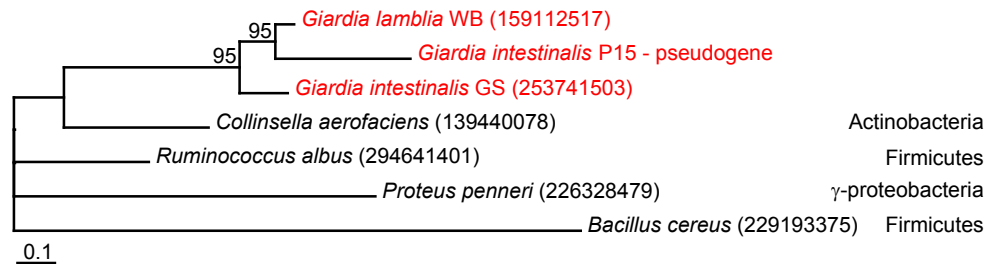

Supplement: Additional file 5 — Evolutionary analyses of a recently acquired Giardia gene. Evolutionary analyses of a recently acquired Giardia gene. (A) A maximum likelihood tree of an acetyl transferase gene (GLP15_874). Only representatives of the homologs showing the highest similarity to the Giardia sequences are included. The tree is based on 139 unambiguously aligned amino acid positions. (B) Alignment of conserved hypothetical proteins from G. intestinalis GS (GL50581_4454) and WB (GL50803_10192) to all detected bacterial homologs. A putative amino acid sequence from a G. intestinalis P15 pseudogene is included and the positions of frameshift and in-frame termination codons are indicated. (C) A maximum likelihood tree based on 137 unambiguously aligned amino acid positions in the alignment shown in (B). Accession numbers are shown in parentheses. Only bootstrap values >50% are shown in the trees. Unambiguously aligned regions were identified manually and removed. Representative homologs with high sequence similarity to these genes were selected using the BLAST-EXPLORER software. [file 1471-2164-11-543-S5.PDF]

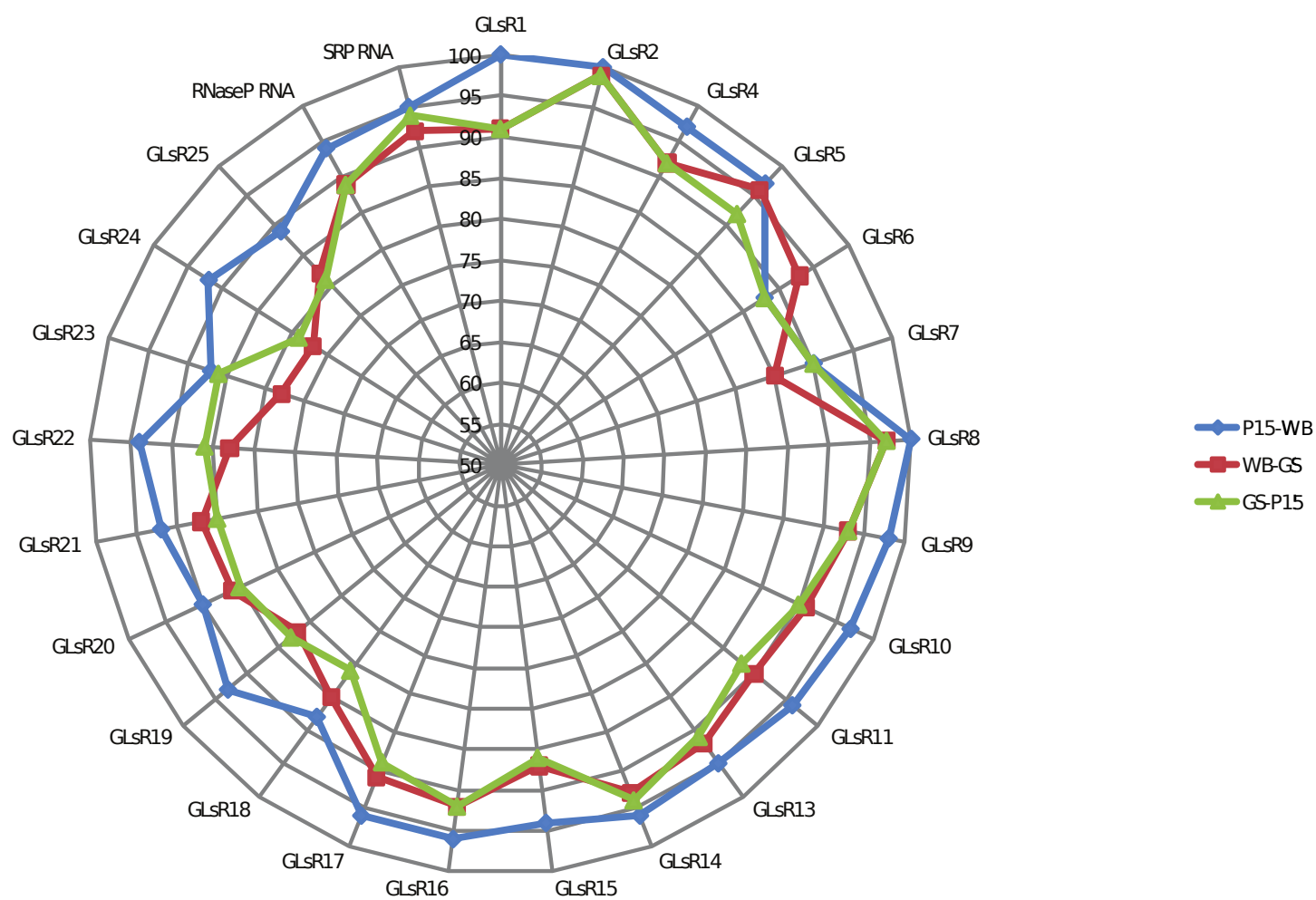

Supplement: Additional file 6 — Non-coding RNA conservation. Conservation of non-coding RNAs across the three Giardia genomes. Non-coding RNAs are generally well conserved, with P15-WB pairs showing the greatest similarity. Examples of ultraconserved non-coding RNAs across all genomes include GLsR2 and GLsR8. [file 1471-2164-11-543-S6.PDF]
