# Supplementary material for: Genome analysis and comparative genomics of a Giardia intestinalis assemblage E isolate
Source: BMC Genomics. 2010 Oct 7;11:543. doi: 10.1186/1471-2164-11-543 (PMC3091692; doi:10.1186/1471-2164-11-543)
Supplement: Additional file 8 — Synteny breaks and PCR primers. Experimental verification of 10 examples of structural variation discovered in the P15 genome. [file 1471-2164-11-543-S8.PDF]

Verification of structural variation in the P15 genome

Structural variation between the P15, WB and GS genomes were identified by manual comparisons of synteny using the Artemis comparison tool (ACT) [1] during annotation of the P15 genome. Ten diverse types of structural variation listed in Table S4 including deletions, insertions, inter- and intra-chromosomal variation were chosen for verification. A screenshot from ACT, including approximate primer positions, primer sequences, expected and obtained PCR products are listed below for each specific example along with a short comment discussing the result of each verification attempt. PCR and sequencing methods can be found in the Material and Methods section.

1. Carver TJ, Rutherford KM, Berriman M, Rajandream M, Barrell BG, Parkhill J: **ACT: the Artemis Comparison Tool**. *Bioinformatics* 2005, **21**:3422-342310.1093/bioinformatics/bti553.

Contig137 : Intrachromosomal rearrangement on chromosome 5

P15contig137-F GTCCCAGGACTTACCAAGGCTAC
P15contig137-R CCTCCAGATATGGCATACAGATGC

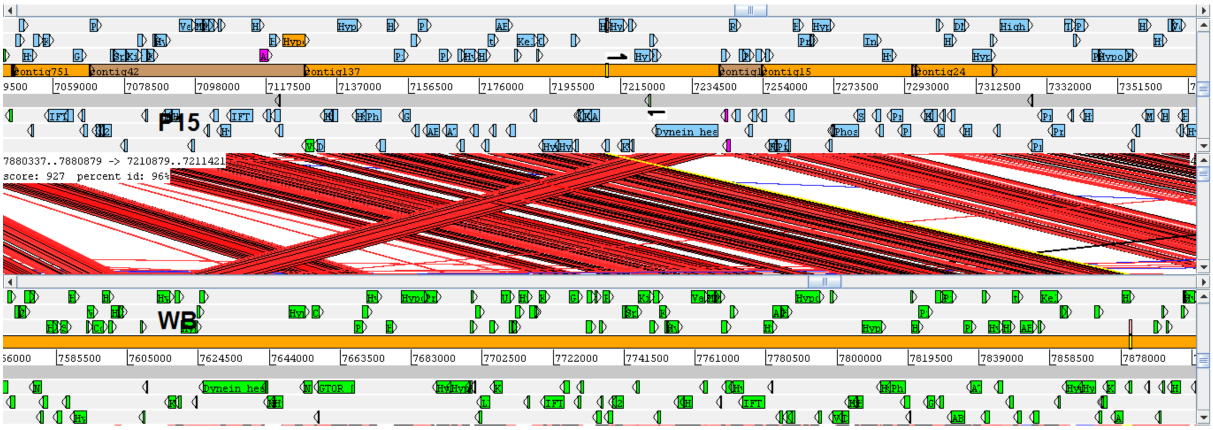

Expected PCR product size: 1406 bp
Estimated PCR product size from gel: ~1400 bp

Comment: Interchromosomal rearrangement on chromosome 5 in P15.

Contig343: Interchromosomal rearrangement between chromosome 1 and 5.

P15contig343-F CCATCACCAGGCGTTAGACTTGTC
P15contig343-R GTGTAGACTCGGAGGACCTGAG

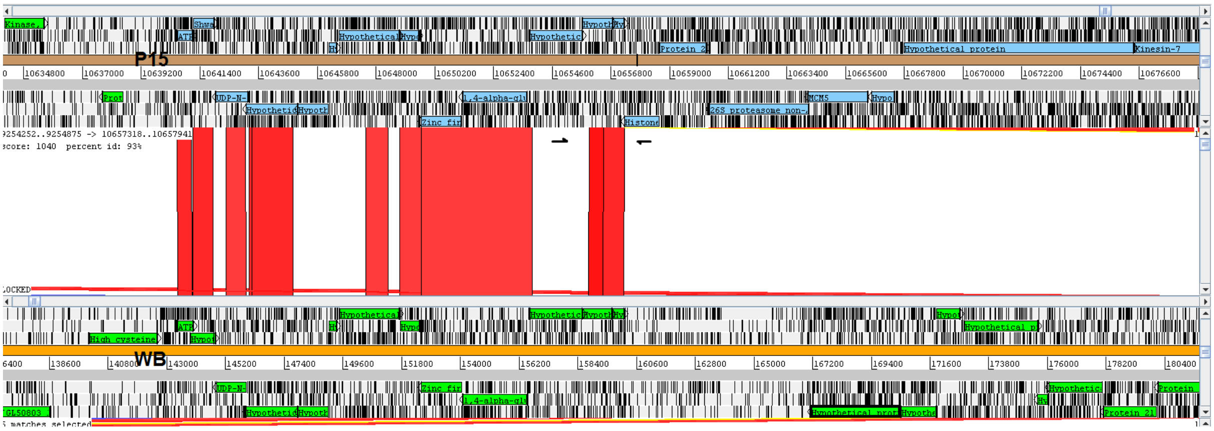

Expected PCR product size: 1046 bp
Estimated PCR product size from gel: ~1100 bp

Comment: Candidate for interchromosomal translocation between chromosomes 1 and 5. Could be some issues with the reverse primer (multiple mapping).

Contig2: Insertion or deletion in WB or P15

P15contig2-F GCATGCTTGCGATTTCTCCCTTAG
P15contig2-R CAGGCTCTGAACATCTGCTTCAGAC

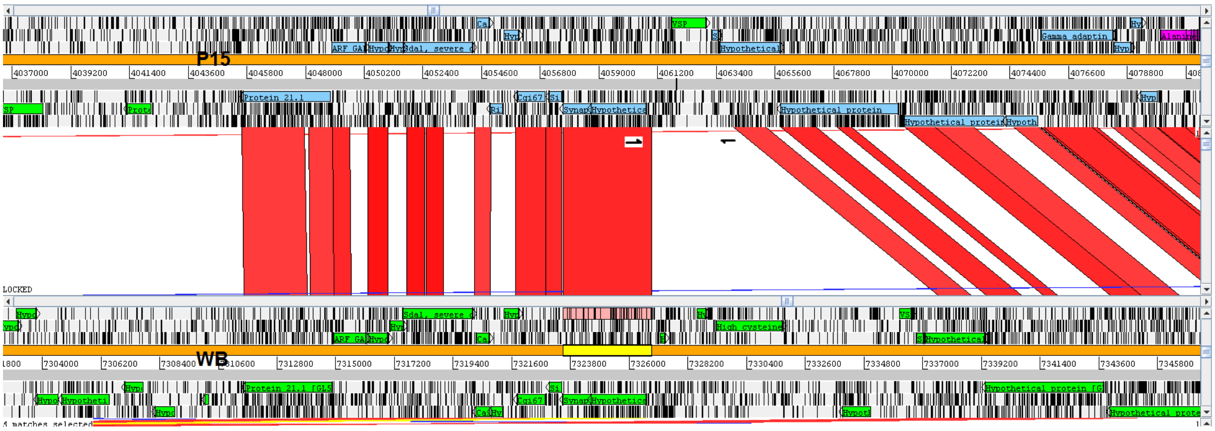

Expected PCR product size: 2570 bp
Estimated PCR product size from gel: none detected

Comment: No PCR product detected, either region is not correctly assembled or there is a large collapsed repeat not traversed in the PCR.

Contig9: Interchromosomal rearrangement between chromosome 1 and 4

P15contig9-F	GTGCAAGGGAAGTGTGGACACG
P15contig9-R	GCAGTCAGCAAGGCACGACAG

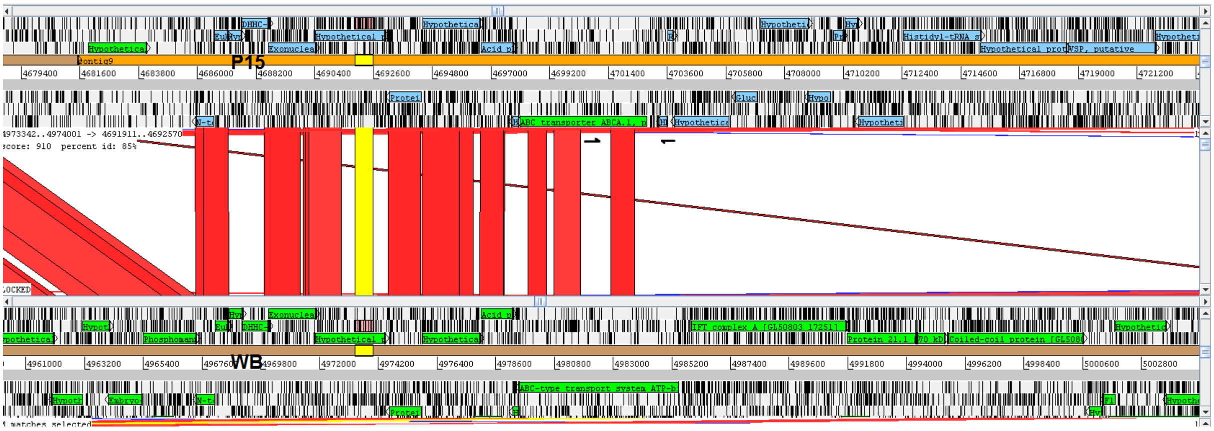

Expected PCR product size: 2457 bp
Estimated PCR product size from gel: ~900 bp

Comment: Misassembly of ABC transporter in P15. In WB no ABC transporter is found next to HRT1-like protein (GL50803_8241), so it appears that a rearrangement has occurred in P15 since the P15contig9-F primer is anchored here.

Contig30: Intrachromosomal rearrangement on chromosome 3.

P15contig30-F	CAGGCTACTGGGAGCAGCAC
P15contig30-R	GACGTGAATCTTTGGCTAATACGGCG

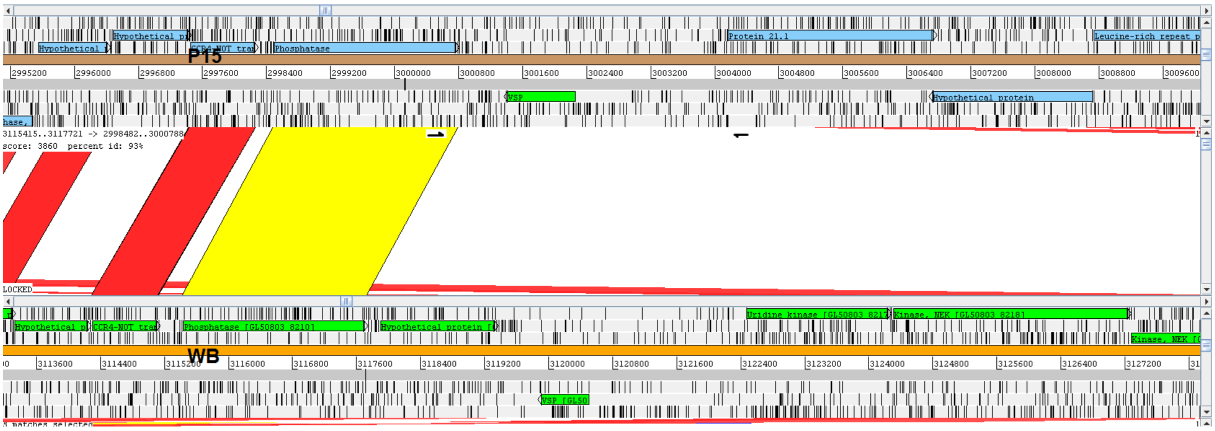

Expected PCR product size: 3503 bp
Estimated PCR product size from gel: ~3000 bp

Comment: Intrachromosomal translocation on chromosome 3.

Contig24: Synteny break in P15

P15contig24-F CAGCTACAGCGCGTTTTCAGAC
P15contig24-R CTAAGGACTCGGTCACACACTCC

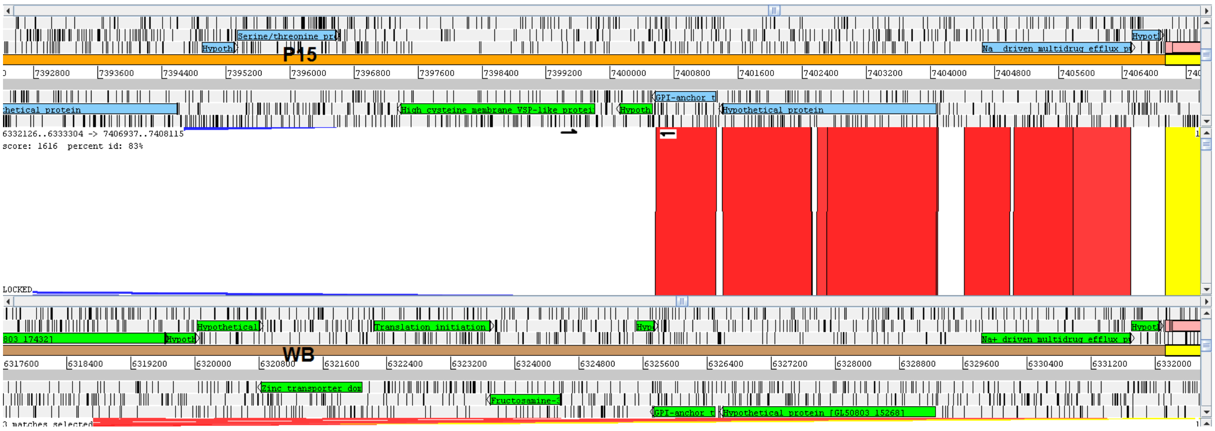

Expected PCR product size: 1363 bp
Estimated PCR product size from gel: 1400 bp

Comment: Intrachromosomal translocation on chromosome 4.

Contig28: Insertion of acetyltransferase

P15contig28-F GCACGGACACAGTGATGTGGTAAG
P15contig28-R GATCAAGGAGCGATCCTCACTACTG

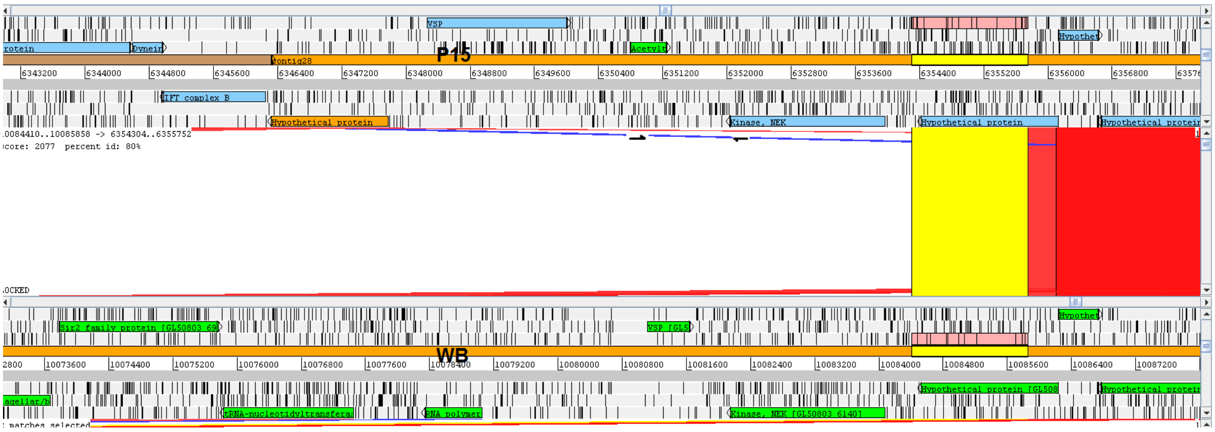

Expected PCR product size: 1380 bp
Estimated PCR product size from gel: 1400 bp

Comment: Confirmation of insertion of the acetyltransferase gene (GLP15_874) next to Kinase, NEK (GLP15_873) on chromosome 5.

Contig45: Invertion and deletion in P15

P15contig45-F	GGTAGATGACCCTTTTCCGTAGAAGAG
P15contig45-R	GAACTGGTCGATACTCTATTTCTCCGTC

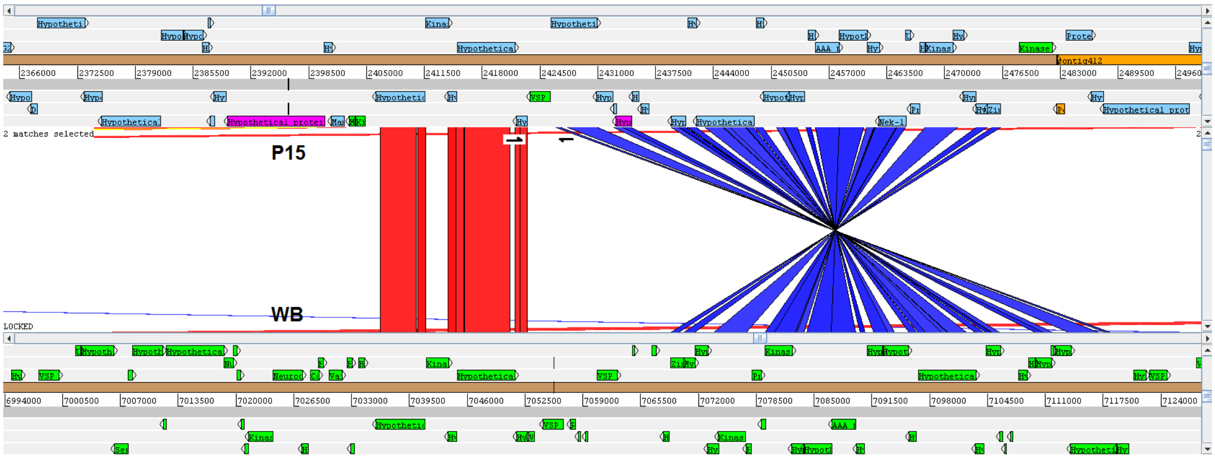

Expected PCR product size: 2881 bp
Estimated PCR product size from gel: ~3000 bp

Comment: Inversion of large region in P15 syntenic to chromosome 4 accompanied by loss of VSP island. Only one VSP remain in P15.

Contig88: Putative deletion in P15

P15contig88-F	GTATCCGCGGGAATATACTCTGACC
P15contig88-R	CCTCTGCAGCTAACATTGGCCTC

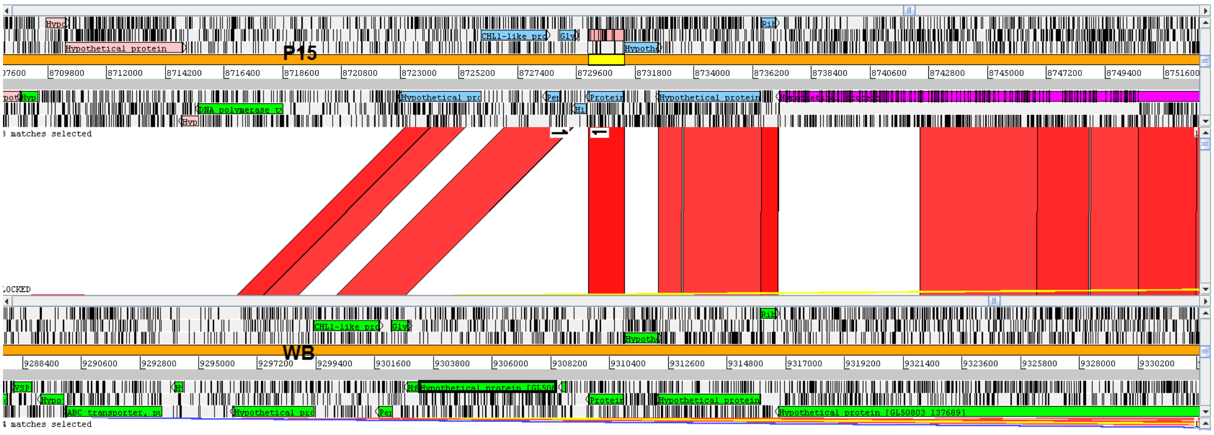

Expected PCR product size: 1080 bp
Estimated PCR product size from gel: ~7000-8000 bp

Comment: Probably a collapsed repeat of the Histone H2B, PCR with primers placed in similar positions in WB would yield a ~7kb PCR product. Also, there is a 7kb fragment of this region as a separate contig in P15 (contig100).

Contig377: Synteny break

P15contig377-F CCAGTTGTGTGTGCAACGAGG
P15contig377-R CGTTGAAAGTTCAGAGTCGCAGG

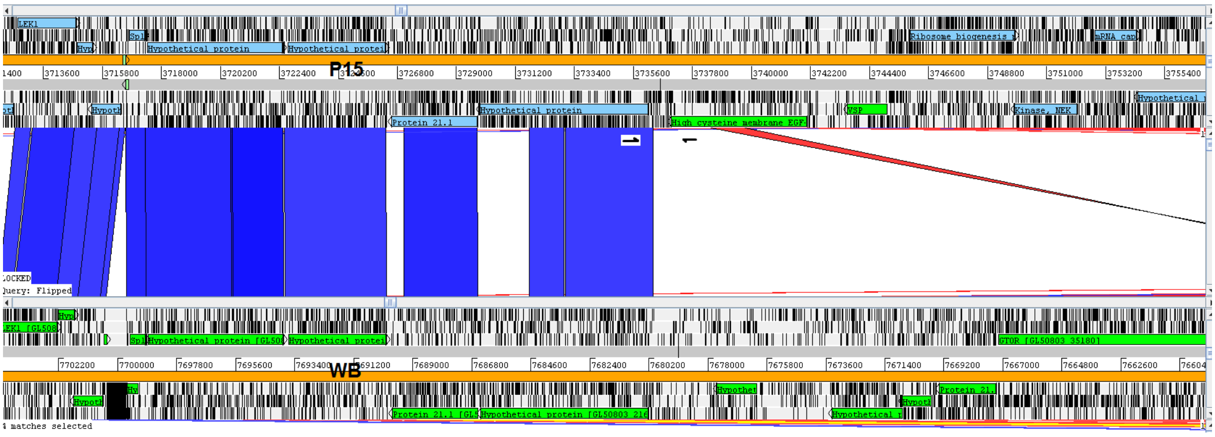

Expected PCR product size: 1416 bp
Estimated PCR product size from gel: ~1400 bp

Comment: Candidate chromosomal translocation between chromosome 3 and 5. Could be problems with repetitiveness of the forward primer (in the HCMP protein).
