# Supplementary material for: Genome analysis and comparative genomics of a Giardia intestinalis assemblage E isolate
Source: BMC Genomics. 2010 Oct 7;11:543. doi: 10.1186/1471-2164-11-543 (PMC3091692; doi:10.1186/1471-2164-11-543)

A

Extended polyadenylation sequence analysis downstream of VSPs

Isolate	VSP 3'UTR sequences (200 bp)	Sequences with motif	Position (relative to stopcodon)	Sequence Logo of motif predicted by MEME
WB	184	183	0-15 bp	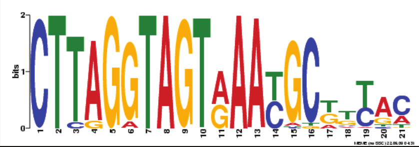
P15	99	99	0-12 bp	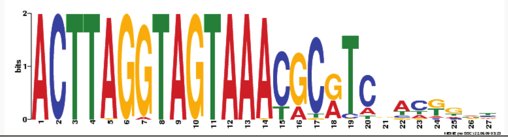
GS	11	11	0-9 bp	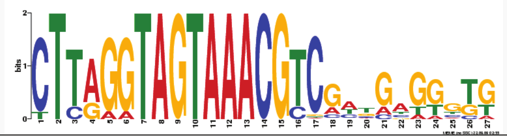

B

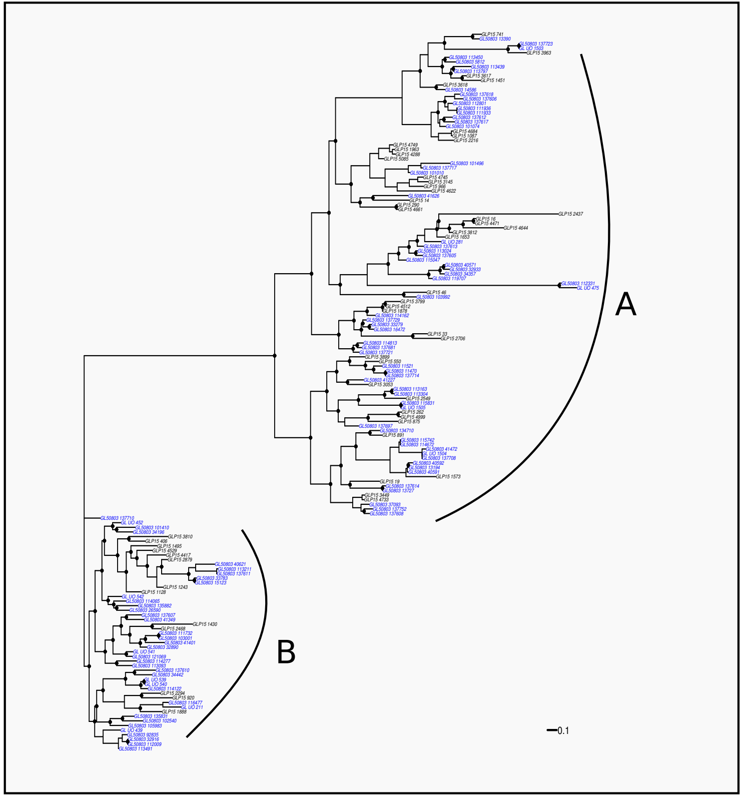

Supplement: Additional file 7 — VSP extended polyadenylation signal and VSP phylogenetic tree. (A) Comparative analysis of the extended polyadenylation sequence of VSP genes. The conservation of the VSP polyadenylation motif was investigated using the MEME software. Downstream regions (200 bp) of VSP genes were extracted and queried for the presence of conserved motifs. The VSP genes of all three Giardia isolates carries a very similar conserved motif that extends over at least 16 bp and is strictly positioned 0-15 bp from the translational stop codon. The P15 motif is more strictly conserved and carries a few more semi-conserved positions for a longer motif than the corresponding motifs in WB and GS. (B) A phylogenetic tree of VSP genes from P15 and WB. The VSP phylogenetic tree is based on a clustalw2 protein alignment of 156 VSP genes from WB and P15. VSP genes were selected for alignment by all-against-all BLASTP, and only VSP genes that showed at least 65% protein identity to the best-matching non-identical hit were used for the alignment (n = 156). In order to improve the alignment, sequences were trimmed (190 aa were removed from the 5' part of all sequences). Subsequent clustering was performed using maximum likelihood as implemented in the software RAxML version 7.0.4 using with the rapid bootstrap setting (-f a). The tree was rendered using the ape package for R. Black dots represent nodes with > = 50% bootstrap support. The gene id (genbank locus tag) is indicated at the tip of the branch. Blue labels represent genes from WB while black labels represent genes from P15. The tree revealed two primary groups (A and B), which are distinguished based on their distance in the tree. [file 1471-2164-11-543-S7.PDF]
